# BCL9 regulates CD226 and CD96 checkpoints in CD8^+^ T cells to improve PD-1 response in cancer

**DOI:** 10.1038/s41392-021-00730-0

**Published:** 2021-08-20

**Authors:** Mei Feng, Zhongen Wu, Yan Zhou, Zhuang Wei, Enming Tian, Shenglin Mei, Yuanyuan Zhu, Chenglong Liu, Fenglian He, Huiyu Li, Cao Xie, Joy Jin, Jibin Dong, Dehua Yang, Ker Yu, Junbin Qian, Diether Lambrechts, Ming-Wei Wang, Di Zhu

**Affiliations:** 1 Department of Pharmacology, School of Basic Medical Sciences and School of Pharmacy, Fudan University, Shanghai, China; 2 School of Pharmacy, Fudan University, Shanghai, China; 3 Key Laboratory of Systems Biology, Innovation Center for Cell Signaling Network, CAS Center for Excellence in Molecular Cell Science, Institute of Biochemistry and Cell Biology, Shanghai Institutes for Biological Sciences, Chinese Academy of Sciences, Shanghai, China; 4 Clinical Translational Research Center, Shanghai Pulmonary Hospital, School of Life Science and Technology, Tongji University, Shanghai, China; 5 School of Medicine, University of California at San Francisco, San Francisco, CA USA; 6 Department of Gynecologic Oncology, Women’s Hospital, Zhejiang University School of Medicine, Hangzhou, China; 7 VIB Center for Cancer Biology and Laboratory for Translational Genetics, Department of Human Genetics, KU Leuven, Leuven, Belgium; 8 School of Life Science and Technology, ShanghaiTech University, Shanghai, China; 9 The National Center for Drug Screening and the CAS Key Laboratory of Receptor Research, Shanghai Institute of Materia Medica, Chinese Academy of Sciences (CAS), Shanghai, China; 10 Shanghai Engineering Research Center of Immune Therapy and Key Laboratory of Smart Drug Delivery, Fudan University, Shanghai, China

**Keywords:** Cancer microenvironment, Cancer genomics, Tumour immunology

## Abstract

To date, the overall response rate of PD-1 blockade remains unsatisfactory, partially due to limited understanding of tumor immune microenvironment (TIME). B-cell lymphoma 9 (BCL9), a key transcription co-activator of the Wnt pathway, is highly expressed in cancers. By genetic depletion and pharmacological inhibition of BCL9 in tumors, we found that BCL9 suppression reduced tumor growth, promoted CD8^+^ T cell tumor infiltration, and enhanced response to anti-PD-1 treatment in mouse colon cancer models. To determine the underlying mechanism of BCL9’s role in TIME regulation, single-cell RNA-seq was applied to reveal cellular landscape and transcription differences in the tumor immune microenvironment upon BCL9 inhibition. CD155-CD226 and CD155-CD96 checkpoints play key roles in cancer cell/CD8^+^ T cell interaction. BCL9 suppression induces phosphorylation of VAV1 in CD8^+^ T cells and increases GLI1 and PATCH expression to promote CD155 expression in cancer cells. In The Cancer Genome Atlas database analysis, we found that BCL9 expression is positively associated with CD155 and negatively associated with CD226 expression. BCL9 is also linked to adenomatous polyposis coli (APC) mutation involved in patient survival following anti-PD-1 treatment. This study points to cellular diversity within the tumor immune microenvironment affected by BCL9 inhibition and provides new insights into the role of BCL9 in regulating CD226 and CD96 checkpoints

## Introduction

Colorectal cancer (CRC) is the third most commonly diagnosed cancer worldwide.^1^ In the last few years, significant new insights into the molecular pathways underlying CRC have provided several new therapeutic options.^2–4^ However, despite the advances in chemotherapeutic and combined targeted treatment options, most patients with metastatic CRC still exhibit poor survival. As such, there is still an unmet need for more effective treatments.^5,6^ Recently, new therapeutic strategies that reinvigorate the immune response directed towards cancer cells were developed. Some of these have successfully paved their way to the clinic, for instance, immune checkpoint inhibitors directed against programmed cell death protein 1 (PD-1) or cytotoxic T lymphocyte-related protein 4 (CTLA-4) are effective against microsatellite-unstable CRC.^7^ Nevertheless, significant challenges remain, especially for microsatellite-stable CRC, which is characterized by a poor response to these checkpoint inhibitors.^7^

The Wnt signaling pathway is a tightly regulated and receptor-mediated transduction pathway that is involved in embryonic development and adult tissue homeostasis.^8,9^ It plays a prominent role in CRC with 80% of patient samples displaying adenomatous polyposis coli (APC) and β-catenin mutations.^10^ In canonical Wnt signaling, Frizzled receptor activation regulates the expression/intracellular localization of β-catenin (β-cat), which binds to co-activators such as B-cell lymphoma 9 (BCL9) and its homolog, B-cell lymphoma 9-like (B9L), thereby mediating Wnt transcription.^11^ We previously generated a potent and selective inhibitor targeting the interaction between BCL9 and β-cat-hsBCL9_CT_-24, which suppresses cancer cell growth and promotes intratumoral infiltration of cytotoxic T cells by reducing regulatory T cells (Treg).^12^

Wnt signaling further contributes to cancer maintenance by mediating immune evasion and resistance to immunotherapies.^13^ Tumors leverage two main Wnt-mediated mechanisms to subvert surveillance and cytotoxicity of the immune response. The first promotes the differentiation and activity of Treg, while the second minimizes the extent of CD8^+^ effector T cell (Teff) infiltration into the tumor microenvironment.^14–16^ Consequently, activation of β-cat results in T cell exclusion, resistance to immunotherapy, and shortened survival of colon cancer patients. Targeting β-cat to block Wnt signaling is a promising strategy to affect immunosurveillance and prevent tumor initiation and metastasis. Especially, elevated Wnt signaling has been implicated in immunosuppressive phenotypes, including insufficient tumor infiltration of immune cells, upon which clinical response to CTLA-4 and PD-1/programmed cell death-ligand 1 (PD-L1) immune checkpoint inhibitors (e.g., ipilimumab and pembrolizumab) is dependent.^17–20^ Recent reports have shown that combining Wnt inhibitors with immuno-oncology (IO) therapies may have synergistic effects in preventing cancer progression.^12^

CD226 is mainly expressed by monocytes, platelets, T cells, and natural killer (NK) cells,^21–24^ and is regarded as a costimulatory receptor. It has two extracellular Ig-like domains and an intracellular kinase domain that phosphorylates downstream signaling effector after binding to CD155 or CD112 (nectin-2).^25^ CD155 (or poliovirus receptor (PVR)/nectin-like molecule-5 or necl5) is a cell adhesion molecule that is commonly overexpressed in tumors associated with poor outcome.^26^ Cellular events induced by CD155 include promotion of cell adhesion,^21^ increase in cell migration, and reduced intrinsic cell contacts.^27^ Recently, CD155 was reported to also regulate the function of tumor infiltrating lymphocytes (TILs) by directly interacting with both stimulatory and inhibitory signaling pathways in T and NK cells. Indeed, CD155 competitively binds to the costimulatory receptor CD226,^26^ as well as the inhibitory receptors CD96 and TIGIT. Together, these molecules constitute a pathway that is analogous to that of CD28/CTLA-4.^28^ Particularly, after costimulatory signaling is established through CD226, NK cell adhesion and cytotoxicity as well as cytokine secretion are activated.^29^ In contrast, interaction of CD155 with TIGIT and CD96 contributes to a “cold tumor” phenotype that facilitates tumor escape and metastasis, leading to poor outcome.^25^ Overexpression of CD155 in tumor cells was also associated with a reduction in TILs and worse treatment outcome, presumably due to interactions between CD155 and the inhibitory receptors CD96 and TIGIT.^30–32^

In CRC, immune checkpoint inhibitors (ICIs) such as PD-1 and PD-L1 antibodies show poor response, suggesting that other immune checkpoints should be targeted to achieve clinical benefits. In a recent study, CD155 was reported to act as an immune checkpoint ligand for tumor and tumor-associated myeloid cells, thus representing a potential novel ICI target.^33^ Furthermore, it was reported that elevated expression of CD155 in human metastatic melanoma is associated with decreased sensitivity to anti-PD-1 immunotherapy.^34^ It, however, remains to be established whether a combination of PD-1 blockade and downstream activation of CD155-CD226 could create costimulatory cytotoxic signaling in CD8^+^ T cells, and whether this might exert a synergistic therapeutic effect.

In this study, we explored the involvement of Wnt signaling in the tumor immune microenvironment (TIME) by assessing how inactivation of BCL9, which acts as a necessary co-factor of β-cat, affects the activity of tumor infiltrating T cells. Specifically, we hypothesized that by inhibiting BCL9 activity, we could shift the balance of CD226 and CD96 checkpoints towards more cytotoxicity and thereby impede tumor growth.

## Results

### Depletion of *Bcl9* inhibits tumor growth by modulating immune cell infiltration

**Fig. 1:**
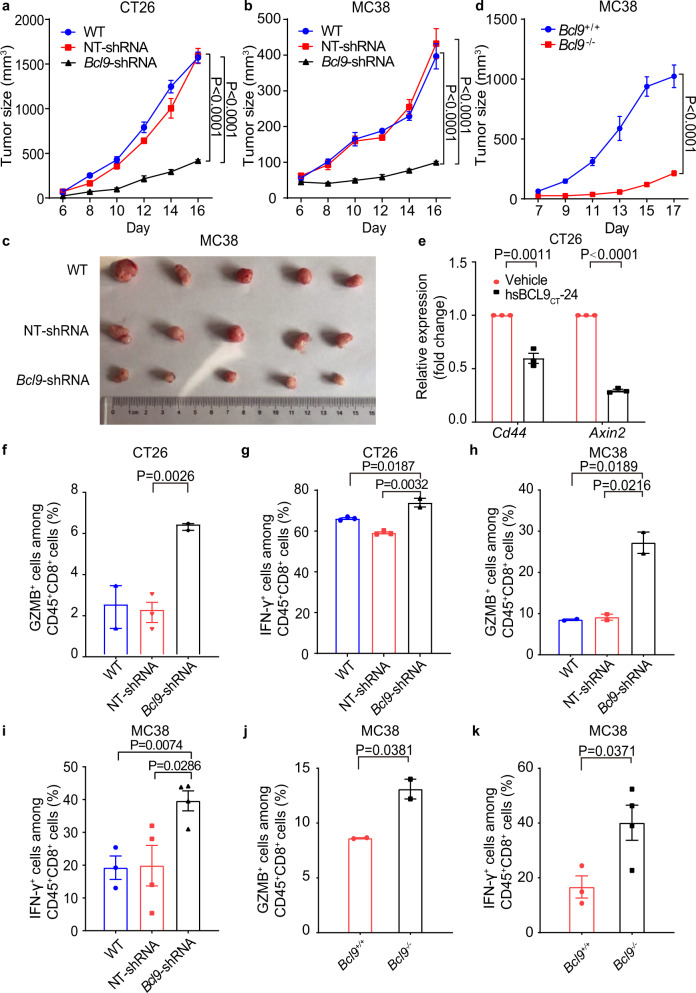
*BCL9* suppression promotes CD8^+^ T cells infiltration. **a** CT26 cells transduced with non-targeting (NT)-shRNA or *Bcl9*-shRNA were inoculated in BALB/c mice (*n* = 5 per cohort). **b** MC38 cells transduced with non-targeting (NT)-shRNA or *Bcl9-*shRNA were inoculated in C57BL/6 mice (*n* = 6 per cohort). **c** Image of tumor tissue from (**c**). **d** Tumor growth in *Bcl9*^+/+^ (wild-type) and *Bcl9*^–/–^ (*Bcl9* knockout) mice injected subcutaneously (*s.c*.) with MC38 cells. **e** qRT-PCR measurement of *Cd44* and *Axin2* expression in CT26 tumor tissue treated with hsBCL9_CT_-24 (*i.p*., 25 mg/kg) or vehicle. **f** Percentage of GZMB^+^ cells among CD45^+^CD8^+^ T cells in tumors from (**a**) was analyzed. **g** Percentage of IFN-γ^+^ cells among CD45^+^CD8^+^ T cells in tumors from (**a**) was analyzed. **h** Percentage of GZMB^+^ cells among CD45^+^CD8^+^ T cells in tumors from (**b**) was analyzed. **i** Percentage of IFN-γ^+^ cells among CD45^+^CD8^+^ T cells in tumors from (**b**) was analyzed. **j** Percentage of GZMB^+^ cells among CD45^+^CD8^+^ T cells in tumors from (**c**) was analyzed. **k** Percentage of IFN-γ^+^ cells among CD45^+^CD8^+^ T cells in tumors from (**c**) was analyzed. Results were denoted as means ± SEM for experiments performed in triplicate. Each experiment was repeated three times, and the statistical significance of differences between groups was determined by Two-way ANOVA or non-parametric test (One-tailed or Two-tailed). *P* < 0.05 means statistically significant.

To characterize the function of *BCL9* (the human gene name) during CRC growth, we designed a shRNA lentivirus plasmid vector pGIPZ to deplete *Bcl9* (the mouse gene name) expression in murine CRC cell lines. Specifically, we depleted *Bcl9* in the MC38 and CT26 cell lines (supplementary Fig. 1a, b), as these express high β-catenin levels and are characterized by Wnt/β-cat dependent growth.^35–37^ Tumor growth in mice subcutaneously bearing CT26 or MC38 cells infected with *Bcl9*-shRNA was significantly suppressed compared to wild-type (WT) mice: tumor growth inhibition (TGI) rate of 73.8% by day 16 in the CT26 model and 83.9% by day 16 in the MC38 model. Compared to NT-shRNA, CT26 or MC38 tumors infected with *Bcl9*-shRNA exhibited a TGI of 74.1% and 85.2% by day 16, respectively (Fig. 1a, b, and c).

Previously, we used the hsBCL9_CT_-24 inhibitor to block interaction between BCL9 and β-cat, and showed that tumor growth inhibition ratio was higher in immune competent than immune compromised mice, suggesting that Bcl9 expressed by immune cells also mediates the therapeutic effect of *Bcl9* knockdown.^12^ We, therefore, also analyzed MC38 tumor growth in WT and *Bcl9*^–/–^ full knockout mice. MC38 cells continued to grow progressively in WT mice, while growth was attenuated in *Bcl9*^–/–^ mice (TGI of 80.5% on day17; Fig. 1d). Wnt downstream-signaling markers such as *Cd44* and *Axin2* were reduced in CT26 or MC38 tumors infected with *Bcl9*-shRNA (supplementary Fig. 1c, d), as well as in tumors treated with hsBCL9_CT_-24 (compared to vehicle-treated tumors; Fig. 1e), suggesting that BCL9 suppression exhibits robust anti-tumor effects by targeting oncogenic Wnt signaling.

To investigate whether *Bcl9* depletion affects tumor immune infiltration, we characterized immune cells derived from CT26 and MC38 tumors implanted in immunocompetent mice by flow cytometry. In *Bcl9*-depleted CT26 tumors, the ratio of tumor-infiltrated Treg was significantly decreased (Supplementary Fig. 1e, while the proportions of cytotoxic granzyme B (GZMB)^+^CD8^+^ T cells, IFN-γ^+^ CD8^+^ T cells, and effector CD8^+^ T cells were increased (Fig. 1f, g, Supplementary Fig. 1f), implying that *Bcl9* depletion inhibits immunosuppressive immune cells. In *Bcl9*-depleted MC38 tumors, cytotoxic CD8^+^ T cells were also significantly increased (Fig. 1h, i), while the ratio of tumor-infiltrated Treg cells were decreased (Supplementary Fig. 1g). Similarly, in *Bcl9*^–/–^ mice injected *s.c*. with MC38 cells, the ratio of tumor-infiltrated Treg cells was significantly decreased (Supplementary Fig. 1h), and the proportion of cytotoxic CD8^+^ T cells was increased (Fig. 1j, k). Overall, it appears that *Bcl9* depletion, either in tumor or stromal cells, not only reduces tumor growth, but also promotes infiltration of cytotoxic and effector CD8^+^ T cells.

### *Bcl9* depletion combined with PD-1 blockade improves the TIME

**Fig. 2:**
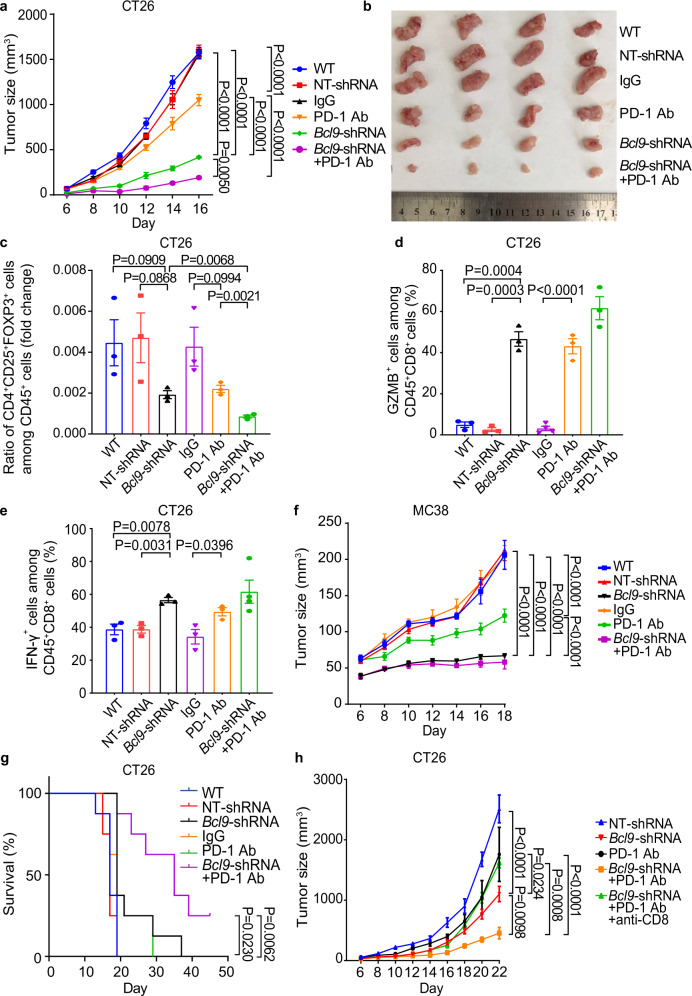
Inhibition of *BCL9* enhances response to anti-PD-1 antibody in CRC models. **a** Combination treatment of *Bcl9*-shRNA and anti-PD-1 antibody resulted in almost complete regression of the CT26 tumor. BALB/c mice were either inoculated with CT26 cells via single flank implantation and treated with immunoglobulin G (IgG), anti-PD-1 antibody [twice-weekly (BIW)], or with CT26 cells that transduced with non-targeting (NT)-shRNA or *Bcl9*-shRNA. The mice were subsequently treated with anti-PD-1 antibody as indicated after tumor volume reached 40–60 mm^3^ (*n* = 5 per cohort). **b** Image of tumor tissue from (**a**). **c** Ratio of CD4^+^CD25^+^FOXP3^+^ cells among CD45^+^ cell populations in the tumors from (**a**). **d** Percentage of GZMB^+^ cells among CD45^+^CD8^+^ T cells in the tumor from (**a**) was analyzed. **e** Percentage of IFN-γ^+^ T cells among CD45^+^CD8^+^ T cells in the tumor from (**a**) was analyzed. **f** Mice inoculated with *Bcl9*-shRNA-transduced-MC38 cells and treated with anti-PD-1 antibody resulted in almost complete regression of the MC38 tumor. C57BL/6 mice were inoculated with MC38 cells via single flank implantation and treated with immunoglobulin G (IgG) or anti-PD-1 antibody (BIW), or with *Bcl9*-shRNA-transduced-MC38 cells and treated with anti-PD-1 antibody as indicated after tumor volume reached 40–60 mm^3^ (*n* = 5 per cohort). Ab, antibody. Results were denoted as means ± SEM for experiments performed in triplicate. **g** Kaplan–Meier analysis of survival end point (tumor size >2000 mm^3^, *n* = 8 per cohort). **h** Mice were inoculated with *Bcl9*-shRNA-transduced-CT26 cells used anti-PD-1 antibody resulted in almost complete regression of the CT26 tumor. BALB/c mice were inoculated with CT26 cells via single flank implantation and treated with anti-PD-1 antibody (BIW), the mice were inoculated with *Bcl9*-shRNA-transduced-CT26 cells used anti-CD8 antibody (intraperitoneal injection, *i.p*., 300 μg per mouse at days 12, 15, and 19 after tumor cell inoculation) plus anti-PD-1 antibody as indicated after tumor volume reached 40–60 mm^3^ (*n* = 5 per cohort). Each experiment was repeated three times, and the statistical significance of differences between groups was determined by Two-way ANOVA or non-parametric test (Two-tailed). *P* < 0.05 means statistically significant.

We then examined whether *Bcl9* depletion has a synergistic effect with anti-PD-1 on tumor growth. Mice inoculated with *Bcl9*-shRNA infected in CT26 tumors were treated with a mouse anti-PD-1 monoclonal antibody. Tumor growth was significantly reduced in *BCL9*-depleted tumors treated with anti-PD-1 compared to anti-PD-1, with a TGI of 81.8% by day 16 (Fig. 2a, b). Flow cytometry of CT26 tumors revealed that anti-PD-1 treatment further increased the abundance of cytotoxic GZMB^+^CD8^+^ T cells and IFN-γ+ CD8^+^ T cells, but decreased the Treg cells (Fig. 2c, e). We also examined the combination of *Bcl9* depletion and anti-PD-1 in MC38 tumors compared to NT-shRNA tumors and confirmed that depletion of *Bcl9* decreases tumor size in response to anti-PD-1, with a TGI of 87.1% by day 18 (Fig. 2f). Finally, combination of *Bcl9* depletion and PD-1 blockade also improved response and survival rates in the CT26 mouse model (Fig. 2g). Overall, these results suggest that depletion of *Bcl9* combined with anti-PD-1 treatment can further increase T cell cytotoxicity and effector function in the TIME.

To directly assess the role of T cells in reducing tumor growth in *Bcl9*-shRNA tumors during anti-PD-1 treatment, we depleted CD8^+^ T cells using a CD8-neutralizing antibody and subsequently examined the effect of *Bcl9*-shRNA combined with anti-PD-1 on tumor growth. It was found that in CD8^+^ T cell-depleted tumor-bearing mice anti-PD-1 no longer exerted an additional inhibitory effect on tumor growth when combined with *Bcl9* depletion (Fig. 2h). The data imply that *Bcl9* depletion enhances anti-tumoral CD8^+^ T cell-mediated immune reactions, thereby amplifying the response to anti-PD-1 in CRC mouse models.

### T-cell profiling by scRNA-seq after depletion or pharmacological inhibition of *Bcl9*

**Fig. 3:**
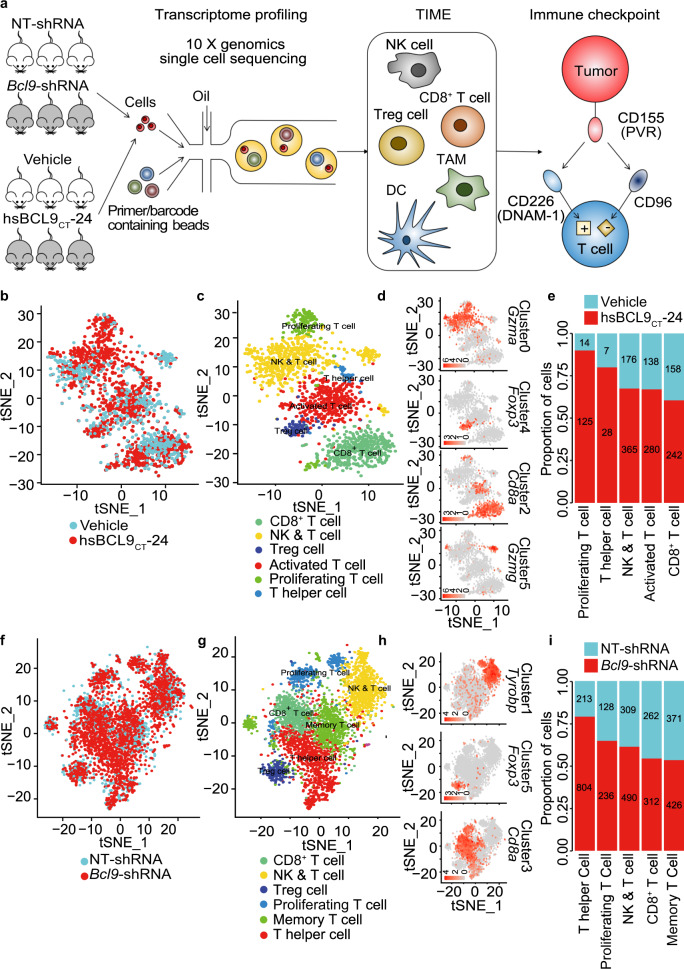
Depletion of *BCL9* changes T-cell cellular landscapes. **a** The workflow of scRNA sequencing. **b** tSNE plot of the tumor sample following treatment with vehicle or hsBCL9_CT_-24 (two groups), color-coded by their associated clusters. **c** Dot plot of the six clusters of T cells from (**b**). **d** tSNE plot of color-coded expression (gray to orange) of marker genes for the clusters from (**c**). **e** The proportion of CD8^+^ T and NK&T cells from 6 samples (vehicle and hsBCL9_CT_-24). **f** tSNE plot of the tumor sample that CT26 cells transduced with non-targeting (NT)-shRNA or *Bcl9*-shRNA (two groups), color-coded by their associated clusters. **g** Dot plot of the seven clusters of T cells from (**f**). **h** tSNE plot of color-coded expression (gray to orange) of marker genes for the clusters from (**g**). **i** The proportion of CD8^+^ T and NK&T cells from 6 samples (NT-shRNA and *Bcl9*-shRNA)

Next, we studied the transcriptional changes in the TIME after *Bcl9* depletion and hsBCL9_CT_-24 treatment using single-cell RNA-seq (scRNA-seq) profiling. CT26 tumors infected with *Bcl9*-shRNA versus hsBCL9_CT_-24, or treated with hsBCL9_CT_-24 versus vehicle, were collected at day 14 and rapidly digested into a single-cell suspension for scRNA-seq using 10x Genomics (Fig. 3a, Supplementary Table 1). Following gene expression normalization for read depth and mitochondrial read count, we obtained high-quality expression data for 95,816 cells (Supplementary Fig. 2a, Supplementary Table 2). After graph-based clustering, 8 cell types were identified based on marker gene expression (Supplementary Fig. 2b, Supplementary Table 3), including cancer cells (*n* = 73,174) by *Wnt10a*, NK&T cells (*n* = 5374) by *Cd3e*, macrophages (*n* = 11,823) by *C1qc*, while classical (*n* = 11823) and plasmacytoid dendritic cells (*n* = 352) (cDCs and pDCs) were identified based on *H2-Aa* and *Klk1b27*, respectively (Supplementary Fig. 2b, Supplementary Table 3). Compared to NT-shRNA tumors, we observed increased proportions of T cells, cDCs, and pDCs in *Bcl9*-shRNA tumors (Supplementary Fig. 2c, d). Similar differences were observed in hsBCL9_CT_-24-treated tumors (Supplementary Fig. 2c, d).

As infiltration of cytotoxic and effector CD8^+^ T cells increased after genetic depletion and pharmacological inhibition of *BCL9*, we investigated the cellular landscape associated with this T cell response in more detail using scRNA-seq data. We subclustered T cells using a similar approach as for the cell types (Supplementary Fig. 3a–d) and observed 6 T cell subclusters in CT26 tumors treated with hsBCL9_CT_-24 (Fig. 3b, c, Supplementary Fig. 3e, f). These included CD8^+^ T cells (marker *Cd8a*), Treg cells (marker *Foxp3*), and NK&T cells (marker: *Klrb1a*, *Gzma*, and *Gzmg*) (Fig. 3c, d, Supplementary Fig. 3e–g, k, l, Supplementary Table 3). Cell fraction analysis showed that CD8^+^ T cells and NK&T cells were increased after hsBCL9_CT_-24 treatment (Fig. 3e). In CT26 tumors infected with *Bcl9*-shRNA and NT-shRNA similar subclusters were identified (Fig. 3f, g, Supplementary Fig. 3h, i) including cytotoxic CD8^+^ T cells (marker *Cd8a*), Treg cells (marker *Foxp3*) and NK&T cells (marker *Tyrobp* and *Klrb1a*) (Fig. 3g, h, Supplementary Fig. 3h, j, m, n and o). Again, CD8^+^ T cells and NK&T cells were increased in *BCL9*-depleted tumors (Fig. 3i). Overall, these result indicate that *BCL9* suppression drives a complex remodeling of infiltrating immune cells, including CD8^+^ T cells and NK&T cells.

### Ligand–receptor interaction identifies the correlation between *CD226-CD155* and *BCL9*

**Fig. 4:**
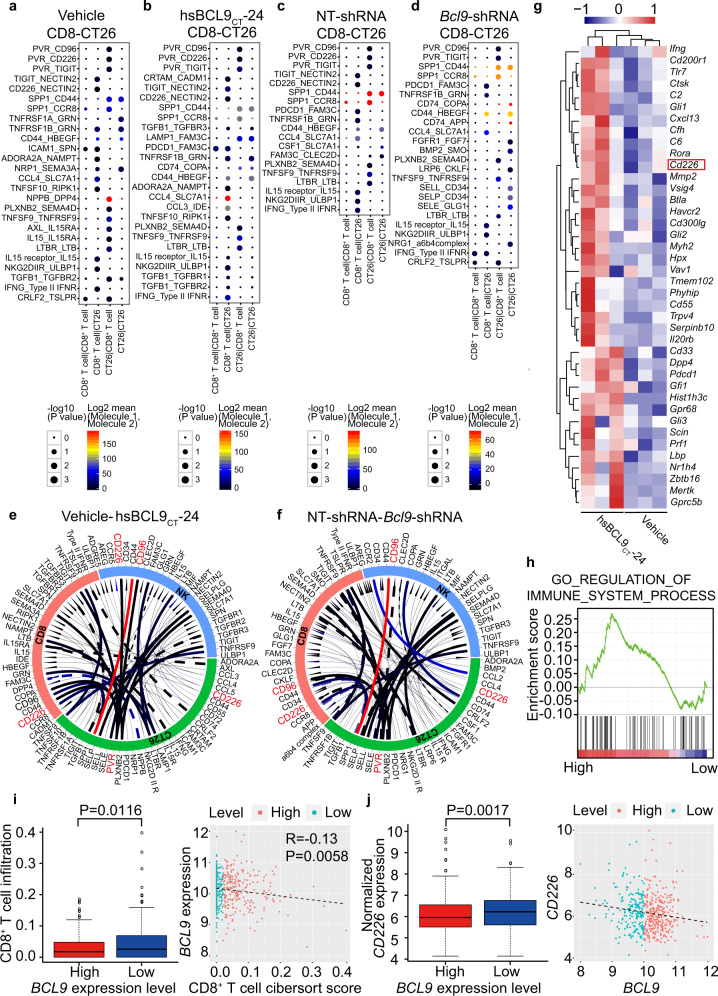
BCL9 is associated with CD155-CD226 checkpoint in CT26-CD8 T^+^ cell interaction. **a** Interaction analysis between CD8^+^ T and CT26 cells in vehicle group. **b** Interaction analysis between CD8^+^ T and CT26 cells in hsBCL9_CT_-24 treated group. **c** Interaction analysis between CD8^+^ T and CT26 cells in NT-shRNA group. **d** Interaction analysis between CD8^+^ T and CT26 cells in *Bcl9*-shRNA group**. e** Circos plots of all of the putative ligand–receptor interactions in vehicle and hsBCL9_CT_-24 treated groups. The color of the line represents the average expression of the ligand–receptor pair in the pair of cells, and the thickness of the line represents the significance of the ligand–receptor enrichment in the pair of cells. The solid line represents the interaction of the hsbcl9_CT_-24 group, and the dotted line represents the interaction of the vehicle group. **f** Circos plots of all of the putative ligand–receptor interactions in NT-shRNA and Bcl9-shRNA groups. The solid line represents the interaction of *Bcl9*-shRNA group, and the dotted line represents the interaction of the NT-shRNA group. **g** Heatmap analysis of CT26 tumor pretreated with vehicle or hsBCL9_CT_-24 (*i.p*., 25 mg/kg). **h** GSEA analysis of CT26 tumor pretreated with vehicle or hsBCL9_CT_-24 (*i.p*., 25 mg/kg). **i S**catter and boxplot analyses of CD8^+^ T cell infiltration associated with *BCL9* expression level in TCGA. **j** Scatter and boxplot analyses of *CD226* expression associated with *BCL9* expression level in TCGA. *P* < 0.05 means statistically significant.

To identify potential interaction between CT26 tumor cells and infiltrating immune cells, we scored ligand–receptor interaction between cancer cells and CD8^+^ T cells by calculating average receptor and average ligand expression in each cell type (Fig. 4a–d). After computing scores for each tumor separately, we averaged them across each tumor model and treatment condition to identify conserved interactions (Fig. 4e, f). Many of the highest-scoring interactions are part of the chemokine family, including TGF-β, IL15, CCL4, and their receptors. Remarkably, CD155/PVR on cancer cells interacted with CD226 expressed in CD8^+^ T cells in all 4 treatment groups, suggesting that this interaction plays a key role in immune modulation.

To further explore this interaction in *Bcl9*-deficient tumors, RNA-seq analyses were performed in CT26 tumor-bearing mice treated with hsBCL9_CT_-24. We found that *Cd226* expression increased significantly compared to vehicle-treated mice (Fig. 4g). In a gene set enrichment analysis (GSEA) of differentially-expressed genes, hsBCL9_CT_-24-treated tumors showed positive correlation with immune regulation (Fig. 4h), consistent with our scRNA-seq findings. Interestingly, T cells were activated and cytotoxic, as indicated by *Prf1* and *Gzmb* that were expressed in CD8^+^ T cells (Supplementary Fig. 4a–d).

Next, we explored the correlation between T cells and the Wnt signaling pathway using CIBERSORT (https://cibersort.stanford.edu) in CRC tumors profiled by The Cancer Genome Atlas (TCGA). We found that tumors with low expression of *BCL9*, *CTNNB1*, and *TCF4* (i.e., tumors with low Wnt pathway activation) contained significantly more infiltrated CD8^+^ T cells (Supplementary Fig. 4e–g). In contrast, tumors expressing high levels of *DVL1, AXIN1*, and *AXIN2* (i.e., high Wnt pathway activation) contained more Tregs (Supplementary Fig. 4h–j). These findings confirm that there is a correlation between *BCL9* and aberrant Wnt pathway activation and CD8^+^ T cell suppression in Colon adenocarcinoma (COAD).

Finally, we evaluated the correlation between *BCL9*, *CD226*, and CD8^+^ T cell infiltration. The fraction of CD8^+^ T cells was significantly lower in COAD when *BCL9* was high (Fig. 4i), while *CD226* was negatively correlated with *BCL9* (*P* < 0.05) (Fig. 4j). Additionally, the abundance of CD8^+^ T cells was increased in COAD when *CD226* was high (*P* < 0.05; Supplementary Fig. 5a). Since *BCL9* was negatively correlated with *CD226* and CD8^+^ T cells in COAD, these findings suggest a correlation between *BCL9* and CD226-positive CD8^+^ T cell infiltration in CRC.

Since expression of the immune checkpoints PD-1 and PD-L1 is correlated with infiltrating immune cells and *Bcl9* depletion is synergistic with anti-PD-1 treatment in mouse tumor models, we studied the relationship among *CD226*, *PD-1* (or *PDCD1*), and *PD-L1* (or *CD274*) in CRC, lung adenocarcinoma (LUAD), lung squamous cell carcinoma (LUSC) and triple-negative breast cancer (TNBC). Both *PD-1* and *PD-L1* were positively correlated with *CD226* in LUAD, LUSC, TNBC and COAD (Supplementary Fig. 5b–i). Triple immunostaining of the murine model of CRC further revealed that CD226 was positive in CD8^+^ T cells, while CD155 was positive in CT26 cells (Supplementary Fig. 5j, k), consistent with the immunofluorescence staining data of the human ovarian cancer revealing that CD155 was co-stained with P53 for cancer cells marker (Supplementary Fig. 5i). Overall, it appears that *CD226* and *PD-1*/*PD-L1* are correlated with multiple cancer types, suggesting that *CD226* activation may compliment anti-PD-L1/PD-1 immunotherapy by activating distinct T cell subsets with synergistic effects.

### *BCL9* associated *APC* mutation is negatively correlated with immunotherapy outcome

**Fig. 5:**
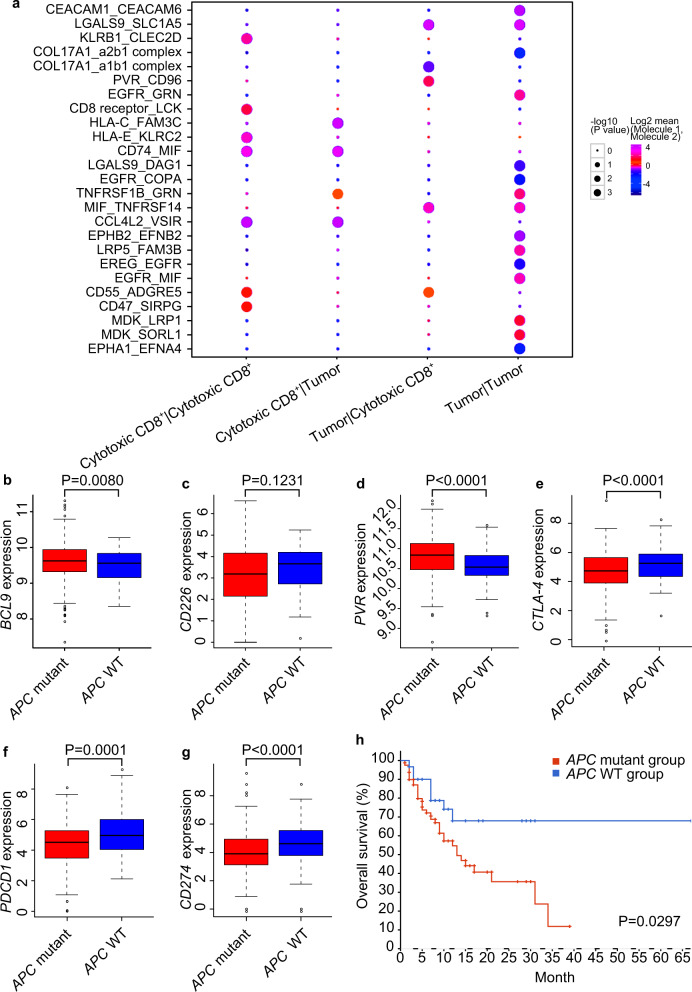
BCL9 and CD155-CD226 are associated with *APC* mutation, which is correlated with patient survival after immune checkpoint inhibitor treatment. **a** Cell–cell interaction in scRNA-seq database for human CRC samples. **b** Boxplot analyses of *BCL9* expression associated with *APC* mutation level in TCGA. **c** Boxplot analyses of *CD226* expression associated with *APC* mutation level in TCGA. **d** Boxplot analyses of *CD155/PVR* expression associated with *APC* mutation level in TCGA. **e** Box plots showing expression differences of *CTLA-4* between *APC* non-mutation and *APC* mutation tumors. **f** Box plots showing expression differences of *PDCD1* (*PD-L1)* between *APC* non-mutation and *APC* mutation tumors. **g** Box plots showing expression differences of *CD274 (PD-L1)* between *APC* non-mutation and *APC* mutation tumors. **h** Correlation of *APC* mutation with immunotherapy outcome in CRC specimens. OS, overall survival. *P* < 0.05 means statistically significant.

To further confirm that the interactions of CD155 with CD226 and CD96 play an important immunoregulatory role in CRC, we quantified cell–cell interaction in a human CRC scRNA-seq dataset. Thirteen patients were enrolled with histopathologically confirmed adenocarcinoma diagnosed by colonoscopy. All tumor tissues were obtained during stage I surgical resection and the samples were rapidly digested to a single-cell suspension and subjected to scRNA-seq. A similar approach as for the mouse CRC models was used to obtain clusters and identify cell types based on marker genes. When assessing cell type interactions between CD8^+^ T cells (marker gene: *CD8A*) and tumor cells (marker gene: *EpCAM*) (Fig. 5a), most of them showed interactions known to be mediating immunity, including that of CD155 with CD96 between CD8^+^ T cells and tumor cells.

In order to investigate the clinical significance of BCL9 and CD155-CD226 in ICI therapy response, we used a cohort of 110 CRC patients from cBioPortal database to analyze the association between gene mutation and overall survival (OS) after treatment.^38^ To further explore the relationship between *APC* mutation and marker genes, we employed the TCGA dataset, and found that *APC* mutation was positively correlated with *BCL9* and *CD155/PVR* expression, but negatively correlated with *CD226* in COAD in TCGA (Fig. 5b–d).^39^ However, expressions of *CTLA-4*, *PD-L1*, and *PD-1* were significantly reduced in *APC* mutant compared to *APC* WT tumors (Fig. 5e–g).

Given the adverse role of mutated *APC* in the immune microenvironment and its effect on *PD-1* expression, patients with *APC* mutation may not benefit from immunotherapy. We analyzed the association between *APC* mutation and immunotherapy outcome using a cohort of CRC patients treated with ICIs showing that they had significantly shorter OS time after immunotherapy than that of *APC* WT patients (Fig. 5h).

### Depletion of *BCL9* modulates CD226-CD155 signaling via phosphorylation of VAV1

**Fig. 6:**
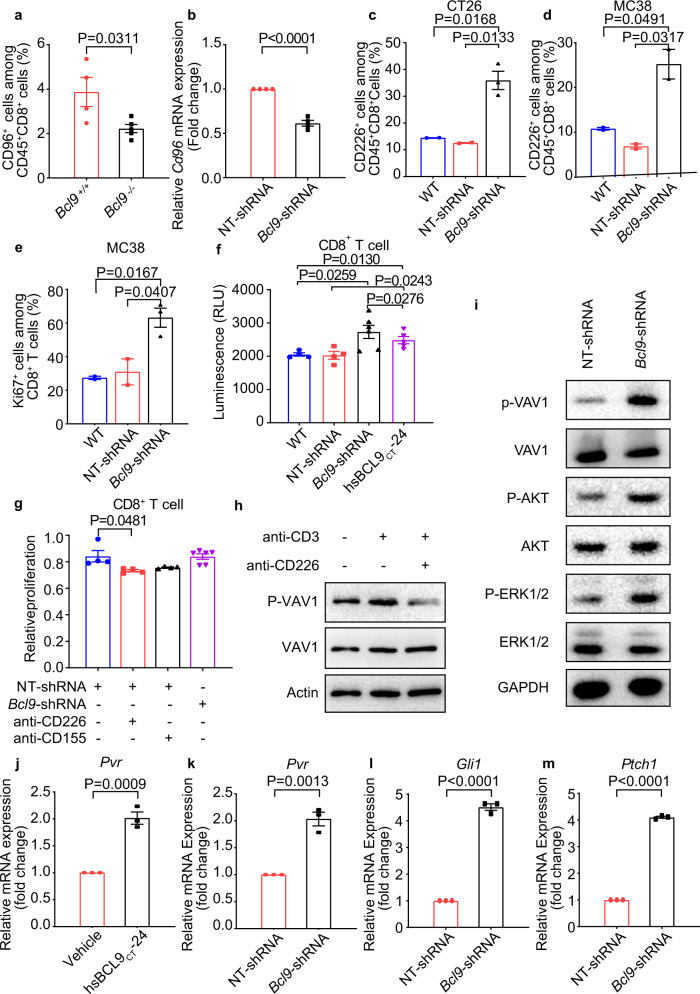
Inhibition of *BCL9* promotes CD155-CD226 checkpoint, which signals via VAV1 phosphorylation. **a** Percentage of CD96^+^ cells among CD45^+^CD8^+^ T cells from MC38 tumor tissue in *BCL9*^+/+^ and *BCL9*^−/−^ mice was analyzed. **b** qRT-PCR measurement of *Cd96* in CD8^+^ T cells treated with NT-shRNA or *Bcl9*-shRNA. **c** Percentage of CD226^+^ cells among CD45^+^CD8^+^ T cells from CT26 tumor tissue in BALB/c mice inoculated with wild-type (WT), NT-shRNA or *Bcl9*-shRNA-transduced-CT26 cells was analyzed. **d** Percentage of CD226^+^ cells among CD45^+^CD8^+^ T cells from MC38 tumor tissue in C57BL/6 mice inoculated with WT, NT-shRNA or *Bcl9*-shRNA-transduced-MC38 cells was analyzed. **e** Percentage of Ki67^+^ cells among CD45^+^CD8^+^ T cells from MC38 tumor tissue in C57BL/6 mice inoculated WT, NT-shRNA or *Bcl9*-shRNA-transduced-MC38 cells was analyzed. **f** Proliferation of CD8^+^ T cell co-cultured with CT26 were treated with *Bcl9*-shRNA or hsBCL9_CT_-24 and analyzed by Cell titer-Glo^®^ Luminescent cell viability assay. **g** Mouse CD8^+^ T cells proliferation was measured after treatment with NT-shRNA or *Bcl9*-shRNA in the presence of anti-CD155 and anti-CD226 antibodies. **h** Immunoblots showing VAV1 and VAV1 phosphorylation on Tyr174 (p-VAV1) in equal amounts of total lysates from CD8^+^ T cells that were treated with or without CD3 antibody (Thermo, 16-0032-81, 0.25 μg) and/or anti-CD226 antibody (Thermo, 0.25 μg) for 5 min. **i** Immunoblots showing phosphorylated VAV1 (p-VAV1), phosphorylated ERK1/2 (p-ERK1/2), phosphorylated AKT (p-AKT) as well as total VAV1, AKT, and ERK1/2 in equal amounts of total lysates from CD8^+^ T cells treated with NT-shRNA or *Bcl9*-shRNA. **j** qRT-PCR measurement of *CD155/Pvr* in CT26 cells treated with hsBCL9_CT_-24 (5 μM) or vehicle for 24 h. **k** qRT-PCR measurement of *CD155/Pvr* expression in CT26 cells transduced with NT-shRNA or *Bcl9*-shRNA. **l** qRT-PCR measurement of *Gli1* expression in CT26 cells treated with NT-shRNA or *Bcl9*-shRNA. **m** qRT-PCR measurement of *Ptch1* expression in CT26 cells treated with NT-shRNA or *Bcl9*-shRNA. Results were denoted as means ± SEM for experiments performed in triplicate. Each experiment was repeated three times, and the statistical significance of differences between groups was determined by non-parametric test. *P* < 0.05 means statistically significant.

CD226 is a transmembrane receptor present on NK and CD8+ T cells. CD155 competitively binds to CD226 and CD96.^26^ The guanine nucleotide exchange factor VAV1 is required for the activation of T cells. Interestingly, upon T-cell activation, the costimulatory molecule CD226 was reported to be one of the most enriched VAV1 interactors.^40^ Significantly less CD96^+^CD8^+^ T cell infiltration was observed in *Bcl9*^–/–^ mice bearing CT26 tumor cells suggesting that *Bcl9* deficiency may promote T cell activation and potentially enhance responsiveness to PD-1 checkpoint blockade (Fig. 6a). Decreased mRNA expression of *Cd96* is also found in *Bcl9*-shRNA treated CD8^+^ T cells compared with NT-shRNA (Fig. 6b), hsBCL9_CT_-24 and vehicle control (Supplementary Fig. 6a). In contrast, depletion of *Bcl9* in CT26 tumor cells increased CD226^+^CD8^+^ T cell tumor infiltration accompanied by increased expression of *Pvr/CD155* (Fig. 6c). In *Bcl9*-depleted MC38 tumors, CD226^+^CD8^+^ T cell tumor infiltration was also increased (Fig. 6d). These results suggest that *Bcl9* suppression promotes CD226^+^CD8^+^ T cell tumor infiltration, but decreases that of CD96^+^CD8^+^ T cells. CD226 was reported to impact CD8^+^ T cell activity and function.^41^ In MC38 tumors, *Bcl9* depletion and knockout both increased T cell proliferation indicted by staining of Ki67 (Fig. 6e, Supplementary Fig. 6b). Interestingly, *Bcl9*-shRNA or hsBCL9_CT_-24 treated CT26 tumor cells co-cultured with CD8^+^ T cells promoted the proliferation of CD8^+^ T cells in vitro (Fig. 6f). We then used CD226 and CD155 neutralizing antibodies in a CD8^+^ T cell proliferation assay. The proliferation of CD8^+^ T cells induced by *Bcl9-*shRNA was abolished by neutralizing antibodies against CD155 and CD226 (Fig. 6g, Supplementary Fig. 6c), suggesting that CD8^+^ T cell proliferation is dependent on CD226-CD155 signaling. Engagement of CD226 is known to elicit tyrosine phosphorylation of VAV1.^40^ Neutralizing antibodies against CD226 indeed decreased the activation of VAV1, as shown by its phosphorylation on Tyr174 (Fig. 6h), implying that engagement of CD226 induces tyrosine phosphorylation of VAV1. Treatment of hsBCL9_CT_-24 or *Bcl9*-shRNA in CD8^+^ T cells increased the level of tyrosine phosphorylation of VAV1, AKT, and ERK, while stimulation of CD8^+^ T cells increased the global quantities of tyrosine phosphorylation (Fig. 6i, Supplementary Fig. 6d). Our results indicate that suppression of *Bcl9* promotes VAV1-AKT-ERK signaling cascades in CD8^+^ T cells.

CD155, expressed on the surface of cancer cells, was shown to promote tumor invasiveness.^42^ Its upregulation in tumor environment–infiltrating myeloid cells restrained anti-tumor immunity by impairing anti-tumor T lymphocyte and NK cell function.^43^ hsBCL9_CT_-24 treatment increased mRNA expression of *CD155/Pvr* in CT26 cells (Fig. 6j), while *Bcl9* depletion in CT26 similarly increased expression of *CD155/Pvr* (Fig. 6k). It was reported that sonic hedgehog activates *CD155* gene expression.^44^ We thus examined whether key components of sonic hedgehog Gli1 (transcription factor) and Patch1 (receptor) were changed. Increased mRNA levels of *Gli1* and *Ptch1* were seen in hsBCL9_CT_-24 or *Bcl9*-shRNA treated CT26 tumor cells (Fig. 6l, m, Supplementary Fig. 6e, f), suggesting that *BCL9* suppression activates *CD155* expression via sonic hedgehog signaling.

### Depletion of *BCL9* inhibits Treg migration

The role of *BCL9* in Treg cells was investigated using the Transwell migration method to simulate tumor infiltration by immune cells. In vitro migration of Treg cells was reduced in *Bcl9*-depleted CT26 cells compared with the NT-shRNA group (supplementary Fig. 7a). Likewise, Treg cells migration in *Bcl9*-depleted MC38 cells was also reduced (Supplementary Fig. 7b). In MC38 cells treated with hsBCL9_CT_-24 decreased migration of Tregs was observed (Supplementary Fig. 7c). When *Ctnnb1* was depleted via shRNA in MC38 cells, Treg cell migration was also reduced, as expected (Supplementary Fig. 7d).

Next, we explored the molecular mechanisms underlying Treg cell migration by examining expression levels of known regulators, including *Ccl4, Ccl22*, and *Tgf-β* using real-time quantitative PCR. *Bcl9* depletion significantly increased *Ccl4* expression and significantly reduced *Tgf-β* expression levels in CT26 cells (Supplementary Fig. 7e, f).^12^ hsBCL9_CT_-24 and CCR4 antagonist C021 were employed to investigate changes in the ability of Tregs to infiltrate tumors. Both inhibitors significantly reduced the tumor infiltration ratio of Treg cells, but interestingly, it did not change the proportion of Treg cells in the spleen (supplementary Fig. 7g, h). This result suggests that Treg cell infiltration is mediated via CCR4 and BCL9/β-cat pathways.

### *BCL9* depletion induces transcriptional differences in CD8^+^ T cells

GSEA was performed to compare T cells in hsBCL9_CT_-24-treated vs. vehicle-exposed CT26 tumors. This revealed that pathways related to IL-2, CD4^+^ T cell memory formation and stimulation of CD8^+^ T were enriched in tumor-infiltrating T cells treated with hsBCL9_CT_-24 (Supplementary Fig. 8a, c). Tumor-infiltrating T cells in *Bcl9*-shRNA compared to NT-shRNA bearing tumors exhibited pathways enriched for TLR, FOXP3, and IL-2 (Supplementary Fig. 8b, d). We found a couple of pathways related to CD8^+^ T cells that are enriched in hsBCL9_CT_-24 and Bcl9-shRNA groups (Supplementary Fig. 9a–f).

Next, transcription factor activity underlying specific mouse CD8^+^ T-cell clusters was analyzed in the scRNA-seq data using SCENIC.^45^ This identified *MYC*, *JUN*, *STAT1*, *JUNB*, and *FL1* as potential transcription factors of CD8^+^ T cells in *Bcl9*-shRNA but not NT-shRNA tumors (Supplementary Fig. 10a). It was found that *MYC* and *STAT1* expression were linked to CD155-related signaling, while downregulation of *Jun*, *Junb*, and *Fli1* were linked to CD226-related signaling, in which *Jun* and *Junb* were identified as CD226 gene promoters (Supplementary Fig. 10a). We then used SCENIC to analyze co-expression of transcription factors and their putative target genes. This identified *Irf1, Junb, Jun, Stat1*, and *Elf1* as candidate transcription factors underlying gene expression differences in T cells (Supplementary Fig. 10b–f).

## Discussion

In this paper, we uncovered that cytotoxic CD8^+^ T cell tumor infiltration was increased by pharmacological inhibition and genetic depletion of *BCL9*. We analyzed the TIME associated with this intervention at single-cell resolution. RNA sequencing technique was applied to characterize CD8^+^ T and NK&T cells and to study their roles in the TIME models. Cellular landscapes and transcriptional features were presented with T, NK, and tumor cells. Key cell clusters and signaling properties among CD8^+^ T and NK&T cells were described and CD155-CD226 was found to be a potential checkpoint regulated by BCL9. We found that CD155/PVR-CD226 signaling occurs through VAV1 phosphorylation and *BCL9* inhibition promotes antibody-mediated PD-1 blockade via promoting cytotoxic CD8^+^ T cell tumor infiltration in mouse models. *BCL9* suppression blocks VAV1 phosphorylation in CD8^+^ T cell as well as increases *GLI1* and *PATCH* expression to promote *CD155* expression in cancer cells (Supplementary Fig. 12). TCGA analysis revealed that *BCL9* upregulation is associated with *APC* mutation.

We for the first time reveal *BCL9* suppression results in altered immune infiltration. Enrichment of CD8^+^ T and NK&T cells in tumor is upon *BCL9* genetic depletion and pharmacological inhibition. BCL9 suppression leads to the engagement of CD226 which in turn activates of VAV1 and promotes CD155 expression possibly via increasing mRNA levels of *Gli1* and *Patch*. BCL9 depletion also significantly decreases *CCL22* expression in tumor cells. Pharmacological inhibition of *BCL9* reduces tumor infiltration by Treg cells due to inhibition of CCR4 expression. Inhibition of Wnt signaling may function through the CCL22-CCR4 axis in Treg cells tumor infiltration.^46–48^

This study is the first demonstration of using scRNA-seq to identify cell clusters (e.g., T cells, macrophages, fibroblasts, endothelial cells, dendritic cells, and granulocytes) in mouse models with *Bcl9* genetic depletion and pharmacological inhibition. We present comprehensive cell distribution map in tumors with genetic depletion of *BCL9* and pharmacological inhibition. Alterations in the proportions of T-cell subpopulations, including decreased percentages of CD8^+^ T cells, tumor-associated macrophages, cancer-associated fibroblasts, tumor endothelial cells, tumor-associated neutrophils, and dendritic cells, indicate a complex TIME in tumors regulated by BCL9. These findings introduce a new way to investigate the role of BCL9 in TIME. Clearly manipulation of cellular microenvironment is of value to dissect functional roles of immune cells of different type in the context of cancer therapeutic outcome evaluation and prognosis prediction.

Immune checkpoint blockade is effective in only a subset of CRC patients with microsatellite instability-high (MSI-H) tumors.^49^ In these patients, the TIME is dynamic during malignant progression. Recent studies have emphasized the role of the TIME in therapeutic resistance in cancer.^50^ Our scRNA-seq study sheds light on the composition of the TIME, which may facilitate the development of novel immunotherapy and enhance understanding about drug resistance in CRC. By identifying differences in immune cell subtypes (e.g., CD8^+^ T and NK&T cells) following BCL9 modulation and CD155-CD226 as a potential checkpoint, our data will certainly help CRC diagnosis and therapy.

BCL9 may be important mainly in *APC* mutated tumors.^51–53^ CRC patients with *APC*^mut^ showed significantly shorter overall survival after ICI immunotherapy than that of *APC* wild-type patients. BCL9 may be negatively correlated with ICI treatment, suggesting inhibition of BCL9 may enhance the outcome of ICI immunotherapy in patients with *APC*^mut^.

There are a number of reports about BCL9 role in non-immune regulation in cancer development. Loss of BCL9/9l was reported to block colonic tumorigenesis and mutations.^51^ Bcl9 and Pygo were found to synergize downstream of Apc to effect intestinal neoplasia in *Apc*^min/+^ mouse models.^53^ BCL9/9L-β-catenin signaling is associated with poor outcome and affects stemness maintenance in colorectal CRC.^54^ Deregulation of BCL9 is an important contributing factor to tumor progression in CRC.^55^ But there is no reports about the role of BCL9 in immune-oncology yet.

CD226 expression is not only associated with PD-1/PD-L1 in CRC, but also in triple-negative breast cancer, lung adenocarcinoma and lung carcinoma suggested by TCGA. It is positively correlated with *APC* mutation, which is an indicator of OS of CRC patients treated with ICIs.^56^ It appears that high infiltration of CD226^+^CD8^+^ T cells might be a survival marker associated with the outcome of ICI treatment in patients of different cancer types.

In the mouse scRNA-seq, we identified that both CD155-CD226 and CD155-CD96 interactions were significantly enriched between CD8^+^ T and CT26 cells while in the human scRNA-seq dataset, only CD155-CD96 was identified. This may be due to the difference of two immune systems and CD226 may plays different roles in the human *vs*. the mouse. Another possibility is that missing CD155-CD226 in human samples is an isolated case because the scRNA-seq data were generated among CRC patients with liver metastasis.

PVR, the ligand for TIGIT, is shared with CD226. TIGIT exerts immunosuppressive action by competing with CD226 for the same CD155 ligand. Blockage of CD226 completely abrogated the effect of TIGIT/PD-L1 in the tumor but did not impact IFN-γ-producing CD8^+^ T cell frequency in the tumor-draining lymph node,^57^ suggesting a unique interplay among CD226, TIGIT and PD-1 in the tumor microenvironment. However, the primary focus of this paper was to study co-stimulatory role of CD226 and co-inhibitory role CD96 in TIME of CRC, respectively.

To investigate the role of Wnt signaling in cancer cells, immune cells, and TIME, receptively, we used three different approaches, including genetic depletion, knockout and pharmacological inhibition. First, in the CT26/MC38 genetic depletion model, CT26 or MC38 cell was *Bcl9* depleted, while the BALB/c and C57BL/6 mice are wild-type. This model shows how *Bcl9* depleted cancer cell impacts on the wild-type CD8^+^ T cell tumor infiltration. Second, in the model of *Bcl9* knockout model, the mice were subcutaneously implanted with wild-type MC38 cell. This model demonstrates how *Bcl9* knockout immune cells were changed in wild-type TIME and how BCL9 driven Wnt signaling alters transcriptional profile of CD8^+^ T cells. Third, in the hsBCL9_CT_-24 treated CT26/MC38 model, hsBCL9_CT_-24, which is a BCL9 and β-cat inhibitor, suppressed transcription of Wnt signaling both in immune cells and cancer cells, without depleting or knockout BCL9 and β-cat protein.

In the CT26 microsatellite stable (MSS) tumor model, anti-PD-1 treatment showed a TGI of 33.88%. Tumor growth was significantly reduced in *Bcl9*-depleted tumors treated with anti-PD-1 antibody, compared to NT-shRNA tumors receiving anti-PD-1 alone, with a TGI of 81.8% by day 16 (Fig. 2a, b), while in the MC38 model with MSI, anti-PD-1 therapy displayed a TGI of 56.72%. We also observed in the same model that depletion of *Bcl9* markedly decreased the tumor size in response to PD-1 antibody, with a TGI of 87.06% by day 18 (Fig. 2f). Our results show that *Bcl9* depletion enhanced the therapeutic effect of anti-PD-1 antibody in both models irrespective of MSS or MSI.

Although our results suggest that targeting the BCL9-CD155-CD226 cascade might have therapeutic implication for CRC, additional preclinical studies are required to validate this hypothesis.

In summary, we found that BCL9 suppression reduced tumor growth, promoted CD8^+^ T cell tumor infiltration, and enhanced anti-PD-1 response. Single-cell RNA-seq revealed that CD226 and CD96 immune checkpoints plays an important role in TIME. Our analyses confirmed many important observations that were previously made by bulk sequencing, either in vitro or in animal models, and highlighted key areas for further studies of BCL9 in the tumor immune microenvironment. These findings would extend our understanding of BCL9 involvement in the tumorigenesis of CRC.

## Materials and methods

### Mouse xenograft model

CT26 *Bcl9*/NT-shRNA cancer cells were cultured as above and harvested for subcutaneously (*s.c*.) inoculation (4 × 10^5^ cells in PBS) in the right flank region of BALB/c female mice (purchased from Charles River) at 6–8 weeks of age. MC38 *Bcl9*/NT-shRNA cancer cells were cultured as above and harvested for *s.c*. inoculation (1 × 10^6^ cells in PBS) in the right flank region of C57BL/6 female mice (purchased from Charles River) at 6–8 weeks of age. *BCL9*^–/–^ mice, on a C57BL/6 background and were obtained from Konrad Basler’s laboratory, Switzerland. MC38 cancer cells were cultured as above and harvested for *s.c*. inoculation (1 × 10^6^ cells in PBS) in the right flank region of *BCL9*^–/–^ or WT C57BL/6 female mice. Tumor volume was measured every other day (V = 0.5 ab,^2^ where a and b are the long and short diameters of the tumor, respectively). Tumor growth inhibition rate (TGI %) per dosing group was calculated according to the following formula: TGI% =[1 − (TVi − TV0)/(TVvi − TVv0)] × 100%, in which TVi represents the mean tumor volume of a dosing group on a specific day, TV0 is the mean tumor volume of a dosing group on day 0, TVvi is the mean tumor volume of vehicle group on a specific day, and TVv0 is the mean tumor volume of vehicle group on day 0. All the animal experiments and care protocols were approved by the Animal Care Committee of Fudan University and conformed to the Animal Management Rules of the National Health Commission of the People’s Republic of China.

In the CT26 model survival experiment, mice were grouped into six randomized cohorts (*n* = 8) and given IgG control, anti-PD-1 antibody [10 mg/kg, intraperitoneal (*i.p*.) injection, twice-weekly (BIW)], or a combination arm [*Bcl9*-shRNA + anti-PD-1 antibody] via *i.p*. injection. Tumor size of >2000 mm^3^ was set as the endpoint.

In the CT26 combination experiment (*n* = 5) and MC38 (*n* = 8) model, mice were grouped into six randomized cohorts and given IgG control, anti-PD-1 antibody (10 mg/kg, *i.p*., BIW), or a combination arm (*Bcl9*-shRNA + anti-PD-1 antibody) via *i.p*. injection.

For CD8 depletion, anti-CD8α (YTS169.4, BioXcell BE0117) was injected *i.p*. (300 μg per mouse) at days 12, 15, and 19 after tumor cell inoculation.

### 10x library preparation and sequencing

The single cell suspensions concentration was 700–1200 cells/μl to input and barcode with a 10x Chromium Controller (10x Genomics). Each barcoded cell sample’s RNA was reverse-transcribed to generate libraries with the reagents from 10x genomics Single Cell 5’ Gel Bead Kit according to the manufacturer’s instructions. Finally sequencing was performed with an Illumina Hiseq 3000 according to the manufacturer’s instructions (Illumina).

### RNA isolation and RNA-Seq analysis

We obtained a total of 6 tumor samples of vehicle and hsBCL9_CT_-24 groups, from which we extracted RNA using the Tirol regent above. Samples were sequenced using the BGISEQ-500 platform at The Beijing Genomics Institute (BGI) for Genomics and Bioinformatics. Raw counts were then normalized to fragments per kilobase of transcript per million mapped reads (FPKM). Differential gene expression was performed based on the negative binomial distribution with the DEGSeq package using the default settings (Wald significance test). To identify enriched signaling pathways, we utilized GSEA and GO analysis.

### Quantification and statistical analysis

#### PCA, t-SNE, and UMAP analysis

All cells that had the number of UMI sequences of low-quality were removed. From the remaining cells, the similarity and variability of cells were summarized by principle component analysis (PCA). As a result, the similarity between cells was observed by the aid of PCA. The expression trend of cell genes is proportional to the sample distance. For visualization, the dimensionality of each dataset was further reduced using either the Barnes-Hut t-Distributed Stochastic Neighbor embedding (t-SNE) or Uniform Manifold Approximation and Projection (UMAP) with Seurat functions Run-TSNE and Run-UMAP. The 7 principle components were summarized and visualized by tSNE (t-Distributed Stochastic Neighbor Embedding) using the default settings of the RunTSNE function. We reanalyzed cells from each of these seven cell types separately in order to identify subclusters. The cells were contrasted using the Seurat Find Markers function to identify marker genes for these subclusters.

#### TMB analysis

TCGA COAD patient mutation information and expression data was downloaded from BROAD GDAC Firehose (http://firebrowse.org/). We kept only the primary solid tumor patient samples for the following analysis. TMB score was calculated as follows: total number of truncating mutations * 2.0 + total number of non-truncating mutations * 1.0 Truncating mutations include nonsense, frame-shift deletion or insertion, and splice-site mutations. Non-truncating mutations include missense, in-frame deletion or insertion, and nonstop mutations.^58^

#### GSVA analysis

GSVA analysis of four group samples was shown as the average expression of each gene of the related cells by using the C2 KEGG pathway subclass data in the MsigDB database to obtain the GSVA score of each pathway. In these data, stimulation of CD8^+^ T signaling was enriched in tumor-infiltrating T cells in the hsBCL9_CT_-24 treated tumor compared with vehicle. TLR and IL2 stimulation signaling were enriched in tumor-infiltrating T cells in tumor bearing *Bcl9*-shRNA compared with NT-shRNA.

#### SCENIC analysis

The SCENIC analysis of four group samples were run using the 20-thousand motifs database for RcisTarget and GRNboost (SCENIC version 0.1.5). The input matrix was the normalized expression matrix, output from Seurat.

#### Statistical analysis

SPSS 22.0 for windows (Chicago, IL, USA) was used for data analysis, and statistical significance was determined using a *t*-test. Numerical data processing and statistical analysis were performed with GraphPad Prism 8; values are expressed as means ± SEM. The *P* values were then calculated using unpaired one/two-tailed Student-*t* tests. *P* < 0.05 was considered statistically significant. For analysis of TCGA dataset, A Kaplan–Meier curve was constructed to compare the overall and disease-free survival rates of two groups. Log-Rank *P* value and HR were calculated with SPSS 22.0. Correlation analysis of gene expression in tumor-infiltrating immune cells was conducted with TIMER. Non- parametric test was applied to small cohort.

## Supplementary information

Supplementary information clean version

Supplementary information marked up changes

Supplementary Figures

## Data Availability

Public Data Resources: The TCGA datasets, including COAD and READ, were downloaded from cBioPortal (http://www.cbioportal.org/). Human scRNA-seq data accession number: SUB9924819. All high-throughput data, flow cytometry data, and immunohistochemical data supporting the current study have been deposited in https://figshare.com/s/970ed37d44f9ac3489db. The custom code used in the manuscript is published at https://figshare.com/s/970ed37d44f9ac3489db.
