# Supplementary material for: BCL9 regulates CD226 and CD96 checkpoints in CD8+ T cells to improve PD-1 response in cancer
Source: Signal Transduct Target Ther. 2021 Aug 20;6:313. doi: 10.1038/s41392-021-00730-0 (PMC8379253; doi:10.1038/s41392-021-00730-0)
Supplement: Supplementary file 1 — Supplementary information clean version [file 41392_2021_730_MOESM1_ESM.docx]

Supplementary Materials for

BCL9 regulates CD226 and CD96 checkpoints of CD8+ T cells in colorectal cancer

Mei Feng^1^, Zhongen Wu^1^, Yan Zhou^2^, Zhuang Wei^3^, Enming Tian^1^，Shenglin Mei^4^, Yuanyuan Zhu^1^, Chenglong Liu^1^, Fenglian He^1^, Huiyu Li^1^, Cao Xie^1^, Joy Jin^5^, Jibin Dong^1^, Dehua Yang^2^, Ker Yu^1^, Junbin Qian^6^, Diether Lambrechts^7,*^, Ming-Wei Wang^1,2,9,*^, Di Zhu^1,8,10,*^

Correspondence to: diether.lambrechts@kuleuven.be (D.L.), mwwang@simm.ac.cn (M.W) or zhudi@fudan.edu.cn (D.Z.)

**Materials and Methods**

Cell culture and T cell expansion

Cell lines CT26 and MC38 (ATCC) were cultured according to the supplier’s recommendations. The culture medium was supplemented with 10% fetal bovine serum (FBS,10099-141, Gibco) and antibiotics. Treg cells were isolated by the CD4^+^CD25^+^ Regulatory T Cell Isolation Kit for mice (130-091-041, Applied Miltenyi Biotec). Treg cells were expanded and cultured with the T Cell Activation/Expansion Kit for mice (130-093-627, Applied Miltenyi Biotec) according to the supplier’s instructions. Cells (2×10^6^) were suspended with 20 μl anti-biotin MACSiBead particles in RPMI 1640 (SH30809.01, Applied HyClone), supplemented with 10% FBS (Gibco), interleukin-2 (IL-2) (1000 U/ml, 402-ML-020, Applied R&D Systems) and TGF- (5 ng/ml, 7666-MB-005, Applied R&D Systems), 2 ml per well in a 24-well plate.

CD8^+^ T cells were isolated by the EasySep™ Mouse CD8^+^ T Cell Isolation Kit (STEMCELL, 18953) according to the manufacturer’s instructions. And CD8^+^ T cells were activated and expanded by the T cell Activation/Expansion Kit for mice (130-093-627, Applied Miltenyi Biotec) according to the manufacturer’s instructions. CD8^+^ T cell cultured with RPMI 1640 supplemented with 10% FBS (10099-141, Applied Gibco), interleukin-2 (IL-2) (1000 U/ml, 402-ML-020, Applied R&D Systems).

Lentivirus production and infection

Lentiviruses were prepared according to the manufacturer’s protocol (GIPZ Lentiviral shRNA, Dharmacon). CT26, MC38 and CD8^+^ T cells were infected by *Bcl9*-shRNA lentiviruses according to the manufacturer’s protocol using the Dharmacon GIPZ Lentiviral shRNA Kit (GE Healthcare). The nontargeting (NT) lentiviral shRNA construct expressing a shRNA sequence with no substantial homology to any mammalian transcript served as a negative control. Cells were infected with NT or *Bcl9*-shRNA lentiviruses as described previously BCL9 protein level was evaluated by immunoblotting.

Immunoblotting

Immunoblotting was performed as described previously using the following primary antibodies against: BCL9 (ab37305, Abcam), VAV1 (sc-8039, Santa Cruz), p-VAV1 (sc-135788, Santa Cruz), AKT (4691T, Cell Signaling Technology), p-AKT (4060T, Cell Signaling Technology), ERK1/2 (4695T, Cell Signaling Technology), p-ERK1/2 (4370T, Cell Signaling Technology), Gapdh (60004-l-g, Proteintech) and β-actin (ab8226, Abcam). Each experiment was repeated three times.

Immunofluorescence staining

Cross-sections of CT26 tumor tissues were used for immunofluorescence staining. RKO and COLO320-DM cells were fixed with PFA and permeabilized in 0.1% Triton X-100, then blocked in 10% FCS for 45 min at 37°C. Primary antibodies against BCL9 (22947-1-AP, Proteintech), CD155 (bs-2525R, Bioss), CD226 (AF0087, Affinity), CD8 (GB13429, Servicebio) were diluted according manufacturers’ instructions and incubated with the tissue samples for 1 h at 37°C. An Alexa Fluor 488 conjugated goat anti-rabbit IgG (H+L) antibody (FITC) (33106ES60, YEASEN) diluted at 1:200 was used as secondary antibody for BCL staining. Alexa Fluor 488 conjugated goat anti-rabbit IgG (H+L) antibody (GB25303, Servicebio) or HRP conjugated goat anti-rabbit IgG (H+L) antibody (GB23303, Servicebio) diluted at 1:200 was used as secondary antibody for CD155, CD226 or CD8 staining. The nuclei were stained with DAPI (MA0127, Meilun Biotech). Images were captured by a confocal microscopy (Zeiss).

Multiplexed immunofluorescence (IF) was performed by WiSee Biotechnology. Primary antibodies sequentially and paired with a unique fluorochrome followed by staining with DAPI. The same process was repeated for the following antibodies: anti-CD226(AF0087, Affinity), anti-CD155(81254,CST), and anti-CD8 (BX50036, Biolynx), anti-P53 (BX50190-C3, Biolynx). Add 100 μl DAPI (5 μg/ml, Sigma, USA) to each slide, washed in distilled water and manually cover slipped. Slides were air-dried, and take pictures with Fluorescence imaging scanner system (3DHISTECH). Images were analyzed using Indica Halo software(Indica Lab).

Transwell migration assay

*In vitro* cell migration assays were performed with Transwell chambers (8 mm pore diameter; Corning Costar), as described in our previous studies. Treg cells were expanded as described above and cocultured with CT26 cells. The reported data represent the average of three independent experiments performed in triplicate. Each experiment was repeated three times.

Flow cytometry analysis

At the end of animal experimentation, tumors were cut and digested in a digestion cocktail (collagenase and deoxyribonuclease). Harvested cells in aliquots of up to 1×10^6^ cells per 100 μl were dispensed into fluorescence-activated cell sorting tubes and stained for flow cytometry according to our standard protocol. Tumor cells were stained with CD45-PeCP.Cy5.5 (103132, Biolegend), CD4-FITC (11-0041-85, eBioscience), CD8-APC-eFluor^®^780 (clone 53-6.7, 47-0081-82, eBioscience), CD3–APC-eFluor^®^780 (47-0031-80, eBioscience), FOXP3-allophycocyanin (APC) (17-5773-82, eBioscience), CD25-phycoerythrin (PE) (102008, BioLegend), CD226-APC (128809, Biolegend), CD96-PE (12-0960-80, eBioscience), Ki67-PE (12-5698-82, eBioscience), IFN-γ–PE (12-7311-82, eBioscience), or granzyme B–eFluor^®^ 450 (48-8898-82, eBioscience). Data acquisition was performed with FACS Aria I followed by analyses with FlowJo software (Tree Star). To identify Treg cells, the following gating strategies were used: (i) selection of live single-cell leukocytes [side scatter (SSC)–A, anti-CD45^+^](anti-CD45); (ii) selection of CD4^+^ T cells(anti-CD4); and (iii) selection of CD25^+^FOXP3^+^ Treg cells (anti-CD25, anti-FOXP3)(Supplementary Fig. 11A). To identify Ki67^+^ CD8^+^ T cells, the following gating strategies were used: (i) selection of live single-cell leukocytes [side scatter(SSC)–A, anti-CD45^+^], (ii) selection of CD8^+^ T cells (anti-CD8), and (iii) selection of Ki67^+^ CD8^+^ T cells( anti-Ki67)(Supplemental Figure. 11B). To identify effector CD8^+^ T cells, the following gating strategies were used: (i) selection of live single-cell leukocytes [side scatter(SSC)–A, anti-CD45^+^], (ii) selection of CD8^+^ T cells (anti-CD8), and (iii) selection of effector CD8^+^ T cells (anti-CD62L) (Supplemental Figure. 11C). To identify CD226^+^/CD96^+^ CD8^+^ T cells, the following gating strategies were used: (i) selection of live single-cell leukocytes [side scatter(SSC)–A, anti-CD45^+^], (ii) selection of CD8^+^ T cells (anti-CD8), and (iii) selection of CD226^+^ CD8^+^ T cells (anti-CD226) and CD96^+^ CD8^+^ T cells (anti-CD96) (Supplemental Figure. 11D). To identify cytotoxic CD8^+^ T cells, the following gating strategies were used: (i) selection of live single-cell leukocytes [side scatter (SSC)–A, anti-CD45^+^](anti-CD45); (ii) selection of CD8^+^ T cells (anti-CD8); and (iii) selection of Granzyme^+^ CD8^+^ T cells (anti-Granzyme) and IFN-γ^+^ CD8^+^ T cells (anti-IFN-gamma)(Supplementary Fig. 11E). The investigator was blinded to the group and labeling allocation during the experiment. Each data point was performed in triplicate, and each experiment was repeated three times.

*In vivo* Treg recruitment assay

CT26 cells (4×10^5^) were injected *s.c.* into 6-8 weeks old BALB/c mice (purchased from Shanghai Lingchang Biotechnology) on day 0. The following day, 3×10^6^ CD8^+^ T cells (Stem Cell kit, according to the manufacturer's instructions) from WT mice were injected intravenously if indicated. On day 10 after tumor inoculation, 1×10^6^ CD4^+^CD25^+^ MACS-enriched Treg cells were given intravenously. If indicated, Treg cells were pretreated *in vitro* with hsBCL9_CT_-24 (5 μM for 2 h) or CCR4 inhibitor C021 (5 μM for 2 h) (APE-BIO), whereas untreated control Treg cells were cultured for the same amount time in medium. Forty-eight hours later, the tumor and spleen were collected and processed into single-cell suspensions for subsequent analysis by flow cytometry. Cells were stained for CD45, CD4, CD25 and FOXP3.

RNA isolation, RT-PCR and quantitative real-time PCR

Total RNA was isolated by using TRIzol reagent (Sigma) from 1 x 10^6^ cultured cells. Then total RNA was reverse-transcribed using 5×PrimeScript RT Master Mix Kit (Takara, RR036A) according to the manufacturer’s instructions. Quantitative RT-PCR was performed using SYBR reagents (Takara, RR420A) according to the manufacturer’s instructions. GAPDH was used as internal control. The primer sequences are following: *Cd44* Forward primer, 5’-TCGATTTGAATGTAACCTGCCG-3';*Cd44* Reverse primer: 5'-CAGTCCGGGAGATACTGTAGC-3';*Axin2* Forward primer, 5’-ATGACGGACAGCAGTGTAGATGG-3';*Axin2* Reverse primer: 5'-GGGTTCTCGGGAAATGAGGTAGA-3'; *CD155/Pvr* Forward primer, 5’-GGGTGGGGATATACGTGTGC-3’;*CD155/Pvr* Reverse primer, 5’-GTTCCTCAGATCCTGTTGGGC-3’; *Ccl4* Forward primer, 5’-CTTCTGTGCTCCAGGGTTCTCAG-3’; *Ccl4* Reverse primer, 5’-CTGGCTTGGAGCAAAGACTGC-3’; *Ccl22* Forward primer, 5’-TATCTGCTGCCAGGACTACATCC-3';*Ccl22* Reverse primer: 5'-CTTCTTCACCCAGACCTGCCT-3'; *TGF-β* Forward primer, 5’-CTCCCGTGGCTTCTAGTGC-3'; *TGF-β* Reverse primer: 5'-GCCTTAGTTTGGACAGGATCTG-3'; *Cd96* Forward primer, 5’- TGGGAAGAGCTATTCAATGTTGG-3';*Cd96* Reverse primer: 5'- AGAGGCCATATTGGGGATGATAA-3'; *Gli1* Forward primer, 5’- CCAAGCCAACTTTATGTCAGGG-3';*Gli1* Reverse primer: 5'- AGCCCGCTTCTTTGTTAATTTGA-3'; *Patch* Forward primer, 5’- AAAGAACTGCGGCAAGTTTTTG-3';*Patch* Reverse primer: 5'- CTTCTCCTATCTTCTGACGGGT-3'; *Gapdh* Forward primer, 5’-GAGTGTTTCCTCGTCCCGTAG-3';*Gapdh* Reverse primer, 5’-TCGCTCCTGGAAGATGGTGAT-3'.

Proliferation assay

For proliferation assays, CT26 cells treated NT-shRNA, *Bcl9*-shRNA or vehicle were seeded into 96-well plates (10,000 cells/well) and incubated with or without 1 μM hsBCL9_CT_-24 before adding CD8^+^ T cells (10,000 cells/well). Proliferation of CD8^+^ T cells was measured by Celltiter-Glo^®^ luminescent cell viability assay (Promega) according to manufacturer’s recommendations.

CD8^+^ T cells proliferation was performed as described in our previous studies by bromodeoxyuridine (BrdU) enzyme-linked immunosorbent assay (ELISA) (Roche) according to the manufacturer’s instructions. The *Bcl9*-shRNA-treated CD8^+^ T cells were treated with or without CD155 neutralizing antibody (R&D, MAB6909-SP, 1μg), and CD226 neutralizing antibody (Thermo Fisher, 16-2261-81, 1μg) for 24h. Then BrdU labeling solution was added into cells for 2 to 24 hours at 37°C. The absorbance of the sample was measured at 450 nm using an ELISA reader (reference wavelength 690 nm). Repeat each experiment three times.

Tissue processing

We implanted 12 mice for each xenograft model to harvest the tumor. Each mouse tumor was minced and digested with collagenase P (Roche) and DNase I (Roche) according to the manufacturer’s instructions. Each sample’s suspension was filtered with 40 μm cell strainer (Falcon) and washed with ice cold DPBS. After centrifuging and discarding supernatant, the sample was resuspend with Red Cell Lysis Buffer (Sigma) to remove erythrocytes. Then single cell suspensions of all samples were resuspended in DPBS with 0.04% BSA. Next, 10μl of this cell suspensions was placed into counting slide to determine viability, living cell rate and single cell rate.

Human Tumor Samples

This study was approved by the Ethics Committee of Zhongshan Hospital in Fudan University (Approval #. B2019-067) and also documented in Ministry of Science and Technology of the People's Republic of China (2021BAT0488). Informed consent was obtained from every patient who agreed to provide specimens for research only. Source data are also provided. (https://doi.org/10.6084/m9.figshare.13121792.v2)

**supplementary Fig. 1.**

**BCL9 suppression inhibits Treg cells infiltration.** a, Immunoblots showing BCL9 in equal amounts of total lysates from CT26 cells transduced with or without nontargeting (NT)-shRNA or *Bcl9*-shRNA. b, Immunoblots showing BCL9 in equal amounts of total lysates from MC38 cells transduced with or without nontargeting (NT)-shRNA or *Bcl9*-shRNA. c, qRT-PCR measurement of *Cd44* and *Axin2* expression in CT26 cells treated with nontargeting (NT)-shRNA or *Bcl9*-shRNA. d, qRT-PCR measurement of *Cd44* and *Axin2* expression in MC38 cells treated with nontargeting (NT)-shRNA or *Bcl9*-shRNA. e, Ratio of CD4^+^CD25^+^FOXP3^+^ cells among CD45^+^ cell populations from CT26 tumor tissue in BALB/c mice inoculated with wildtype (WT), NT-shRNA and *Bcl9*-shRNA-transduced-CT26 cells was analyzed. f, Ratio of CD44^+^ CD62L^-^ cells of CD45^+^ CD8^+^ cell populations from CT26 tumor tissue in BALB/c mice inoculated with wildtype (WT), NT-shRNA and *Bcl9*-shRNA-transduced-CT26 cells was analyzed. g, Ratio of CD4^+^CD25^+^FOXP3^+^ cells among CD45^+^ cell populations in the tumors from MC38 tumor tissue in C57BL/6 mice inoculated with wildtype (WT), NT-shRNA and *Bcl9*-shRNA-transduced-MC38 cells was analyzed. h, Ratio of CD4^+^CD25^+^FOXP3^+^ cells among CD45^+^ cell populations in the tumors from MC38 tumor tissue in *BCL9*^+/+^ and *BCL9*^-/-^ mice was analyzed. Results were denoted as means ± SEM for experiments performed in triplicate. Each experiment was repeated three times, and the statistical significance of differences between groups was determined by non-parametric Student’s t test. P < 0.05 means statistically significant.

**supplementary Fig 2.**

**Cellular landscape of pharmacological inhibition and genetic depletion of BCL9. a,** A t-distributed stochastic neighbor embedding (tSNE) analysis of single cells from 12 mouse tumor samples (left), and tSNE analysis of the associated cell types (right). **b,** Expression of marker genes for the cell type defined above each panel. **c,** Fraction of cells originated from each of the 12 samples. **d,** Fraction of cells originated from each of four groups.

**supplementary Fig 3.**

**T cellular landscape of pharmacological inhibition and genetic depletion of BCL9.** **a-b,** tSNE plots of the 12 samples and T cells, color-coded by their associated clusters. **c-d,** UMAP plots of the 12 sample and T cells, color-coded by their associated clusters. **e-f**, tSNE plots of the sample type (6 samples from Fig. 3**b**) and T cells, color-coded by their associated clusters. **g**, Dot plots showing representative marker genes across different subsets in 6 samples from Fig. 3**b**. **h-i**, tSNE plots of the sample type (6 samples from Fig. 3**f**) and T cells, color-coded by their associated clusters. **j,** Dot plots showing representative marker genes across different subsets in 6 samples from Fig. 3**f. k,** tSNE plot of color-coded expression (gray to orange) of *Pclaf* for the clusters3. **l**, tSNE plot of color-coded expression (gray to orange) of *Fcrl6* for the clusters6. **m**, tSNE plot of color-coded expression (gray to orange) of *Spc24* for the clusters4. **n**, tSNE plot of color-coded expression (gray to orange) of *Emb* for the clusters2. **o**, Proportion of cells in NT-shRNA and Bcl9-shRNA.

**supplementary Fig 4.**

**Correlation of BCL9 and Wnt pathway genes with CD8^+^ T and Treg cells.** a-d, tSNE plots of *Prf1* and *Gzmb* expression in four groups (Vehicle_hsBCL9_CT_-24, NT-shRNA_*Bcl9*-shRNA). e-g, CD8^+^ T cells associated with different gene expression levels (*BCL9, CTNNB1* and *TCF4*) in COAD of TCGA. h-j, Treg cells associated with different gene expression levels (*AXIN2, AXIN1* and *DVL1*) in COAD of TCGA. P < 0.05 means statistically significant.

**supplementary Fig. 5**

**The expression of *CD226* is related with *PD-1* and *CTLA-4* in human cancer. a,** Scatter and boxplot analyses of CD8^+^ T cells infiltration associated with *CD226* expression level in TCGA.**b.** Pearson correlation of *CD226* and *PD-1* expression in the LUAD samples. **c,** Pearson correlation of *CD226* and *PD-L1* expression in the LUAD samples. **d,** Pearson correlation of *CD226* and *PD-1* expression in the LUSC samples. **e,** Pearson correlation of *CD226* and *PD-L1* expression in the LUSC sample. **f,** Pearson correlation of *CD226* and *PD-1* expression in the TNBC samples. **g,** Pearson correlation of *CD226* and *PD-L1* expression in the TNBC samples. **h,** Pearson correlation of *CD226* and *PD-1* expression in the COAD samples. **i,** Pearson correlation of *CD226* and *PD-L1* expression in the COAD samples. **j**, Immunofluorescence analysis of CD226 and CD155 signals in the mouse CT26 tumor. **k**, Co-localization of CD226 and CD8 in the mouse CT26 tumor. **l**, Co-localization of p53 and CD155 in the human ovarian cancer. CD226 (red), CD155 (green), CD8 (pink) and DAPI (blue). Scale bar = 10 *μ*m.

**supplementary Fig. 6**

**Molecular mechanism of *BCL9* inhibition promotes CD155-CD226 checkpoint, which signals via VAV1 phosphorylation. a,** qRT-PCR measurement of *Cd96* in CD8^+^ T cells treated with vehicle or hsBCL9_CT_-24(5μM). **b**, Percentage of Ki67^+^ cells among CD45^+^CD8^+^ T cells from MC38 tumor tissue in Bcl9^+/+^ and BCL9^-/-^ mice was analyzed. **c**, Mouse CD8^+^ T cells proliferation was measured after treatment with vehicle or hsBCL9_CT_-24(5μM) in the presence of anti-CD155 and anti-CD226 antibodies. **d**, Immunoblots showing phosphorylated VAV1 (p-VAV1) and total VAV1 in equal amounts of total lysates from CD8^+^ T cells that were treated with vehicle or hsBCL9_CT_-24 (5 μM or 10 μM). **e**, qRT-PCR measurement of *Gli1* expression in CT26 cells treated with vehicle or hsBCL9_CT_-24 (5 μM). **f**, qRT-PCR measurement of *Patch* expression in CT26 cells treated with vehicle or hsBCL9_CT_-24 (5 μM). Results were denoted as means ± SEM for experiments performed in triplicate. Each experiment was repeated three times, and the statistical significance of differences between groups was determined by non-parametric Student’s *t* test. P < 0.05 means statistically significant.

**supplementary Fig. 7**

**Molecular mechanism of BCL9 inhibition in Treg cells migration and tumor infiltration. a,** Migration of freshly isolated Treg cells co-cultured with CT26 cells transduced with NT-shRNA or *Bcl9*-shRNA. WT, wildtype. **b,** Migration of freshly isolated Treg cells co-cultured with MC38 cells transduced with NT-shRNA or *Ctnnb1-*shRNA. **c,** Migration of freshly isolated Treg cells co-cultured with MC38 cells pretreated with hsBCL9_CT_-24 (5 μM) for 24 h. **d,** Migration of freshly isolated Treg cells co-cultured with MC38 cells transduced with NT-shRNA or *Ctnnb1-*shRNA. **e,** qRT-PCR measurement of *Ccl4* expression in CT26 cells transduced with NT-shRNA or *Bcl9*-shRNA. **f,** qRT-PCR measurement of *Ccl22* and *Tgf-β* expression in CT26 tumor tissue transduced with NT-shRNA or *Bcl9*-shRNA. **g** , CT26 cells were engrafted into BALB/c (subcutaneously) followed, 24 hours later, by injection of 3 × 10^6^ CD8^+^ T cells (intravenously) (if indicated), and on day 10 after tumor inoculation, 1 × 10^6^ Tregs (CD4^+^ CD25^+^) were given (intravenously) hsBCL9_CT_-24. Tregs were treated with hsBCL9_CT_-24 for 2 hours in vitro before injection, and C021 Tregs were treated with the CCR4 antagonist C021 for 2 hours before injection. Ratio of CD4^+^CD25^+^FOXP3^+^ cells among CD45^+^ cell populations in the tumor samples was analyzed. **h,** Ratio of CD4^+^CD25^+^FOXP3^+^ cells among CD45^+^ cell populations in the spleen samples from **g** was analyzed. Results were denoted as means ± SEM for experiments performed in triplicate. Each experiment was repeated three times, and the statistical significance of differences between groups was determined by non-parametric Student’s t test. P <0.05 means statistically significant.

**supplementary Fig. 8**

**Gene set enrichment analysis (GSEA) analysis of BCL9 deprived T cell population. a**, GSEA analysis of CD8^+^ T cells in vehicle and hsBCL9_CT_-24 treated groups, using the immunologic signature gene sets database (C7) from the Molecular Signatures Database (MSigDB) , and the normalized enrichment score (NES) ranking is selected for bar plot. **b**, GSEA analysis of CD8^+^ T cells in NT-shRNA and Bcl9-shRNA groups, using the immunologic signature gene sets database (C7) from the Molecular Signatures Database (MSigDB) , and the normalized enrichment score (NES) ranking is selected for bar plot. **c**, GSEA analysis of CD8^+^ T cells in vehicle and hsBCL9_CT_-24 treated groups on the database MSigDB C5: ontology gene sets, and the normalized enrichment score (NES) ranking is selected for bar plot. **d**, GSEA analysis of CD8^+^ T cells in NT-shRNA and Bcl9-shRNA groups on the database MSigDB C5: ontology gene sets, and the normalized enrichment score (NES) ranking is selected for bar plot.

**supplementary Fig. 9**

**Gene set enrichment analysis (GSEA) analysis of BCL9 deprived T cell population. a-f** showed enrichment plots of different clusters.

**supplementary Fig. 10**

**SCENIC analyses of CD8^+^ T cells after genetic depletion of BCL9. a,** SCENIC analysis of CD8^+^ T cells in NT-shRNA and *Bcl9-*shRNA groups. **b-f**, tSNE plots of endothelial cells, color-coded for (left) the expression of *Irf1* (**b**), *Junb* (**c**), *Jun* (**d**), *Stat1* (**e**) and , *Elf1* (**f**), and for (right) the AUC of estimated regulatory activities of these transcription factors, corresponding to the degree of expression regulation of their target genes.

**supplementary Figure 11.**

**FACS analysis of tumor infiltrates.** **a,** Representative FACS plots showing the gating strategy used to identify CD4^+^CD25^+^Foxp3^+^Treg cells. **b,** Representative FACS plots showing the gating strategy used to identify Ki67^+^CD8^+^ T cells. **c,** Representative FACS plots showing the gating strategy used to identify CD44^+^CD62L^-^CD8^+^ T cells. **d,** Representative FACS plots showing the gating strategy used to identify CD96^+^ CD8^+^ T cells and CD226^+^CD8^+^ T cells. **e,** Representative FACS plots showing the gating strategy used to identify Granzyme B^+^CD8^+^ T and IFN-γ^+^ CD8^+^ T cells.

**supplementary Fig. 12**

**Schematic figure illustrating the mechanism of BCL9 inhibition in regulating CD226 and CD96 checkpoints.** CD155-CD226 signaling occurs through VAV1 phosphorylation and BCL9 inhibition promotes antibody-mediated PD-1 blockade via promoting cytotoxic CD8^+^ T cells tumor infiltration, while decreases Treg cells migration and tumor infiltration via *Ccl4*, *Ccl22*, *Tgf-β*.

**supplementary Table 1.**

**Tumor sample information**.

| **ID** | **Age (week)** | **Sex** | **Location** | **Treatment** | **Size (mm)** | **Cell number** |
| --- | --- | --- | --- | --- | --- | --- |
| 5 | 10 | Female | *s.c.* | Vehicle | 13105 | 4907 |
| 8 | 10 | Female | *s.c.* | Vehicle | 12.294.5 | 5376 |
| 10 | 10 | Female | *s.c.* | Vehicle | 13.48.64.3 | 3500 |
| 4 | 10 | Female | *s.c.* | hsBCL9_CT_-24 | 14.34.42.2 | 4443 |
| 12 | 10 | Female | *s.c.* | hsBCL9_CT_-24 | 11.14.62.3 | 5396 |
| 16 | 10 | Female | *s.c.* | hsBCL9_CT_-24 | 13.036.43.2 | 5679 |
| 18 | 10 | Female | *s.c.* | NT-shRNA | 16.58.44.2 | 10984 |
| 19 | 10 | Female | *s.c.* | NT-shRNA | 12.3105 | 11128 |
| 22 | 10 | Female | *s.c.* | NT-shRNA | 11105 | 9660 |
| 24 | 10 | Female | *s.c.* | Bcl9-shRNA | 107.23.6 | 9837 |
| 25 | 10 | Female | *s.c.* | Bcl9-shRNA | 97.43.7 | 13782 |
| 29 | 10 | Female | *s.c.* | Bcl9-shRNA | 8.47.63.8 | 11124 |
|  |  |  |  |  |  |  |

**supplementary Table 2.**

**Cell number in each sample**.

| Cluster | 18 | 19 | 22 | 24 | 25 | 29 | 12 | 16 | 4 | 10 | 5 | 8 |
| --- | --- | --- | --- | --- | --- | --- | --- | --- | --- | --- | --- | --- |
| Total | 10984 | 11128 | 9660 | 9837 | 13782 | 11124 | 5396 | 5679 | 4443 | 3500 | 4907 | 5376 |
| CT26 | 8427 | 9131 | 6700 | 7519 | 10230 | 8466 | 3789 | 3700 | 3694 | 2637 | 3920 | 4961 |
| Fibroblasts | 134 | 81 | 591 | 143 | 93 | 166 | 130 | 95 | 21 | 93 | 53 | 36 |
| Granulocytes | 171 | 214 | 284 | 156 | 316 | 189 | 105 | 132 | 76 | 54 | 53 | 71 |
| Macrophage | 1302 | 1180 | 1519 | 1132 | 1885 | 1324 | 783 | 1191 | 360 | 394 | 592 | 161 |
| Pericytes | 43 | 60 | 116 | 60 | 80 | 68 | 21 | 62 | 30 | 58 | 60 | 36 |
| T cell | 713 | 359 | 303 | 663 | 1009 | 715 | 487 | 373 | 236 | 237 | 184 | 95 |
| Plasmacytoid dendritic cell | 46 | 36 | 36 | 53 | 43 | 62 | 28 | 29 | 0 | 5 | 8 | 6 |
| Classical dendritic cells | 148 | 67 | 111 | 111 | 126 | 134 | 53 | 97 | 26 | 22 | 37 | 10 |

**supplementary Table 3.**

**Cell cluster characteristics**.

| Cluster | Annotated name | Marker genes |
| --- | --- | --- |
| CT26 | CT26 | *Baiap2l1, Rpl39l* |
| Natural killing and T cell | NK&T cell | *Klrb1F, Klrb1A, Ncr1, Tyrobp, Gzmc, Ncam1, Klrd1* |
| B cell | B cell | *Ptprc, Cd79a, Cd19* |
| Macrophage | Macro | *Cd68, Adgre1, Itgam, Csf1r* |
| Cancer-associated fibroblast | CAF | *Col1a1, Col3a1, Col5a1, Fstl1, Bgn* |
| T cell | T cell | *Cd3d, Cd3e, Cd3g* |
| T cell | Activated T cell | *Grap2, Kbtbd11, Cd69, Cd44, Cd25, Il2ra* |
| T cell | CD8^+^ T cell | *Cd8A, Nrn1, Cxcr6* |
| T cell | Proliferating T cell | *Esco2, Brca1, Neil3, Cdc6, Pclaf, Clspn, Hist1H2Ap, Spc24, Rrm2* |
| T cell | Treg cell | *Il2Ra, Izumo1R, Foxp3, Penk, Cd4, Il7Rα, Nrp1* |
| T cell | T helper cell | *Slfn5, Fosl1, Mt1, Cd4, Il7Rα, Cxcr3, Tbx21, St2, Gata3, Ccr6, Cxcr5, Icos, Pdcd1* |
| T cell | Memory T cell | *Ly6C2, Lat, Sell, Klrg1, Cd3* |
