# Supplementary figures and images for: BCL9 regulates CD226 and CD96 checkpoints in CD8+ T cells to improve PD-1 response in cancer
Source: Signal Transduct Target Ther. 2021 Aug 20;6:313. doi: 10.1038/s41392-021-00730-0 (PMC8379253; doi:10.1038/s41392-021-00730-0)

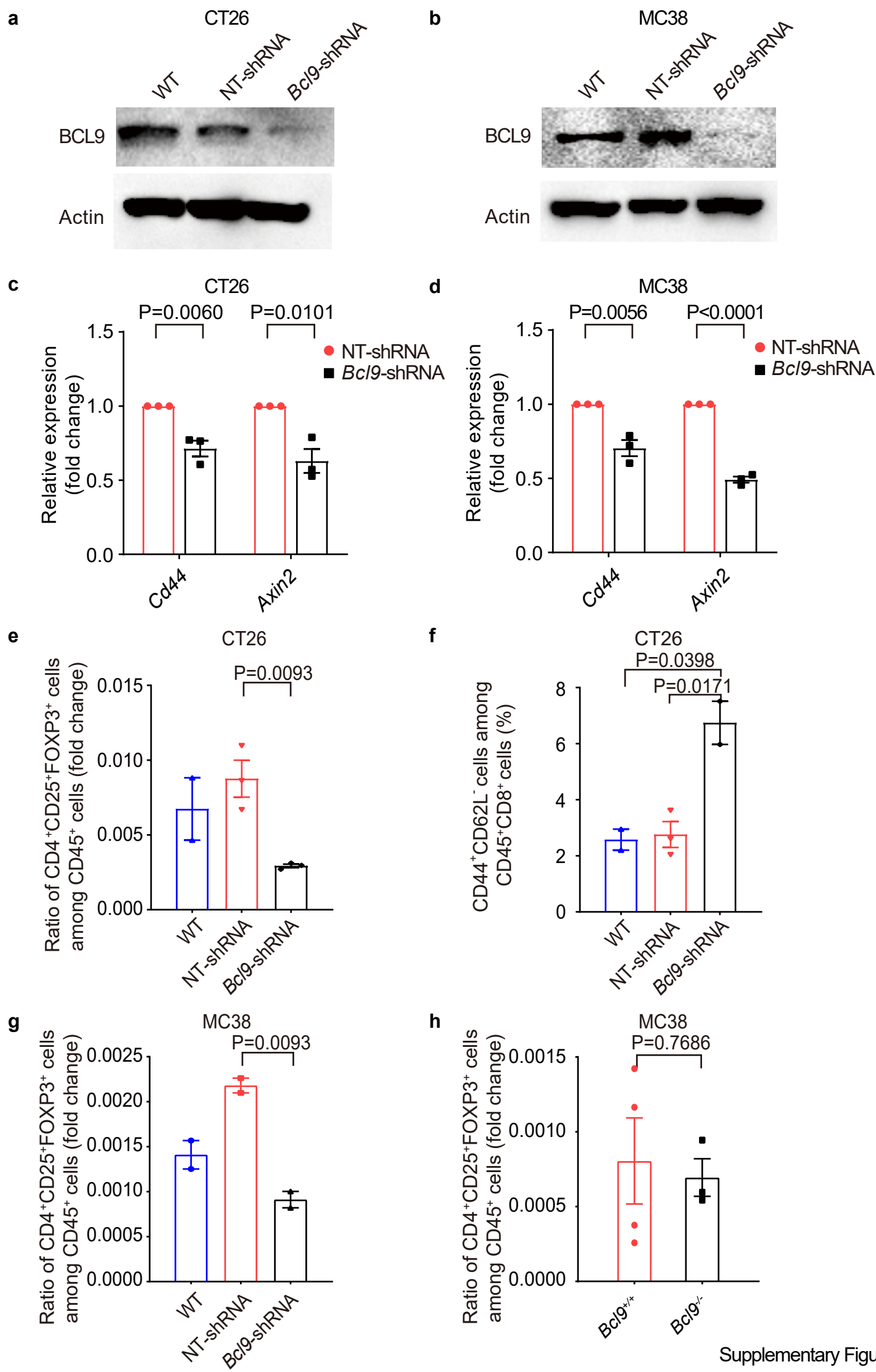

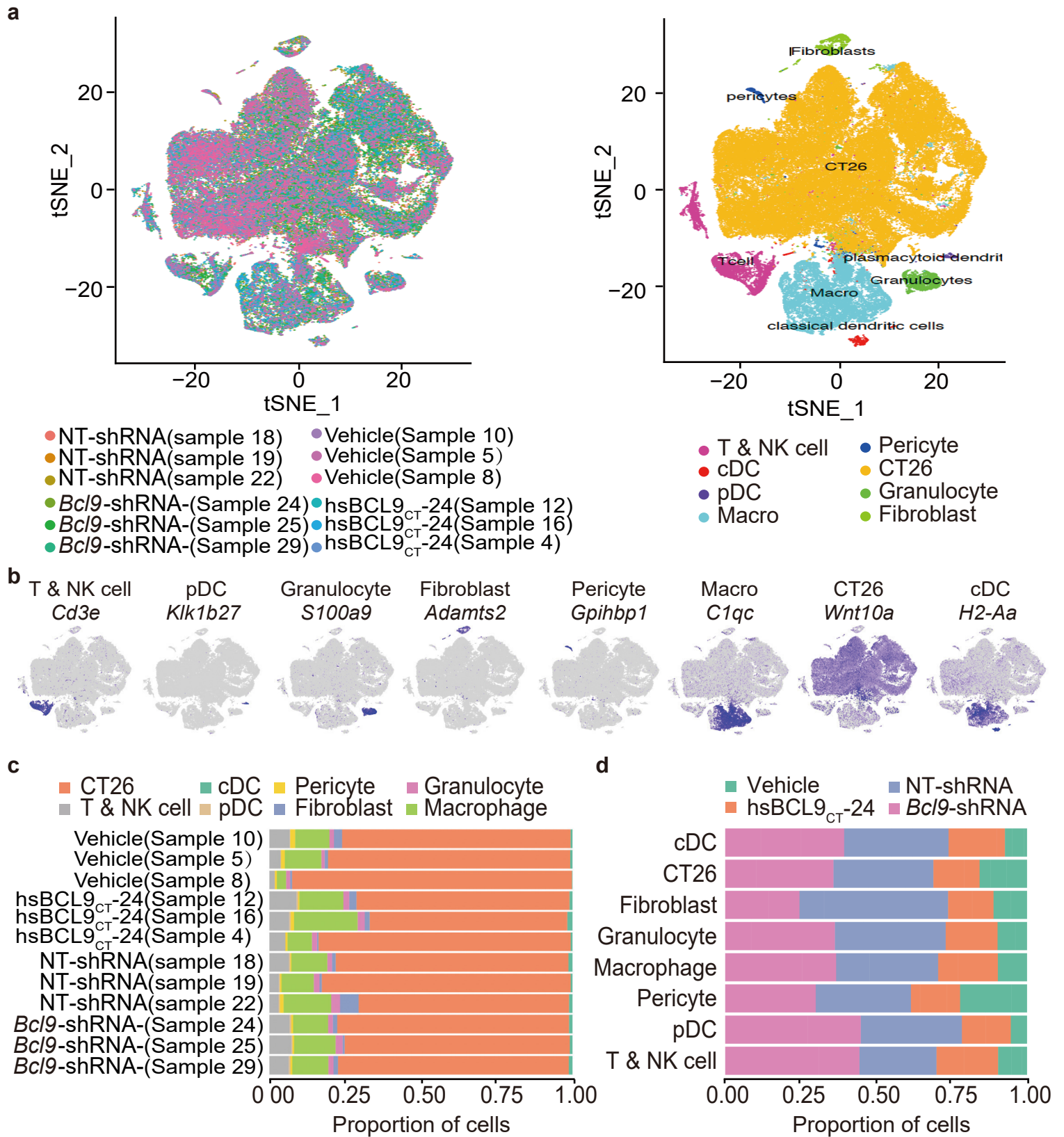

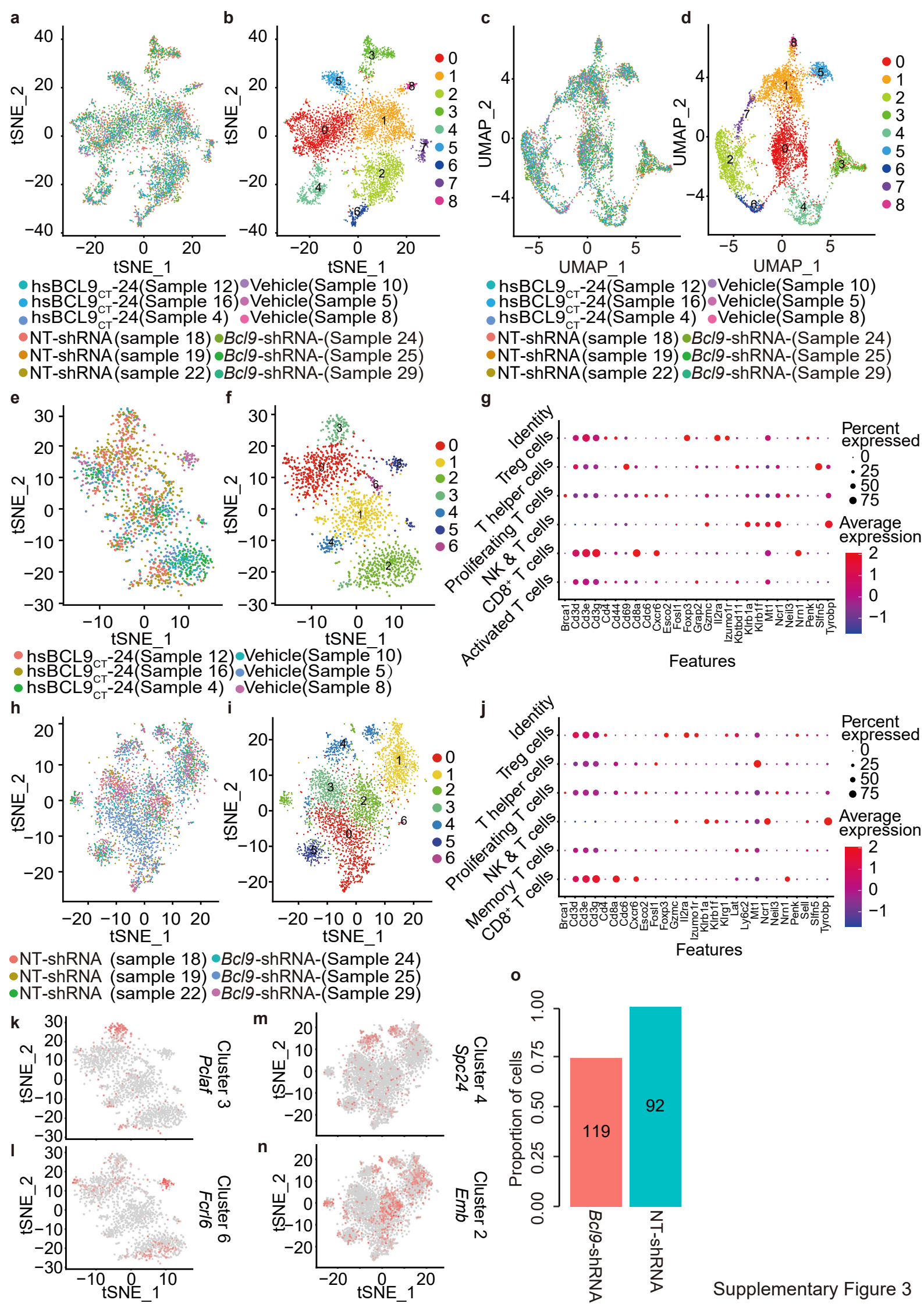

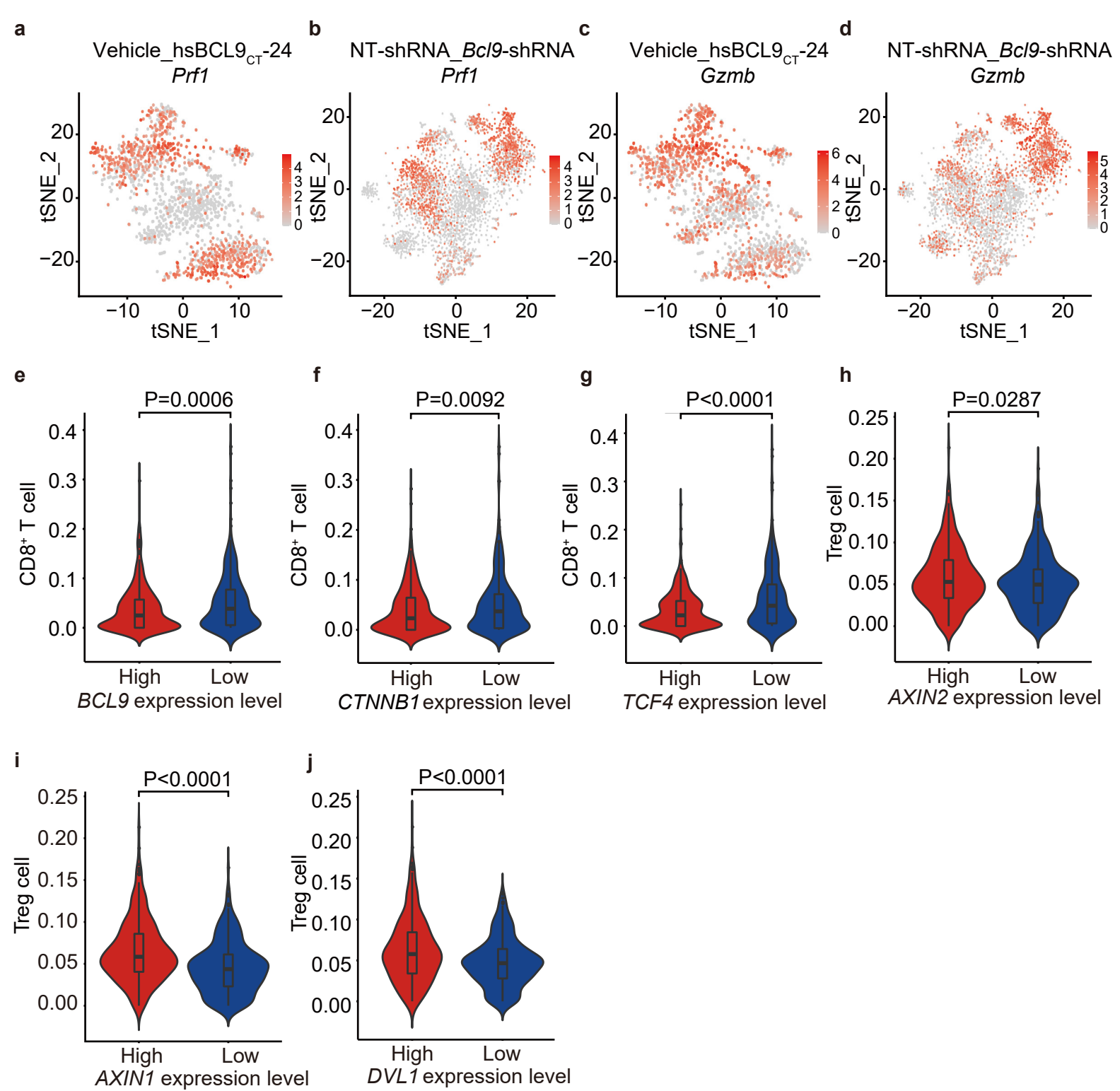

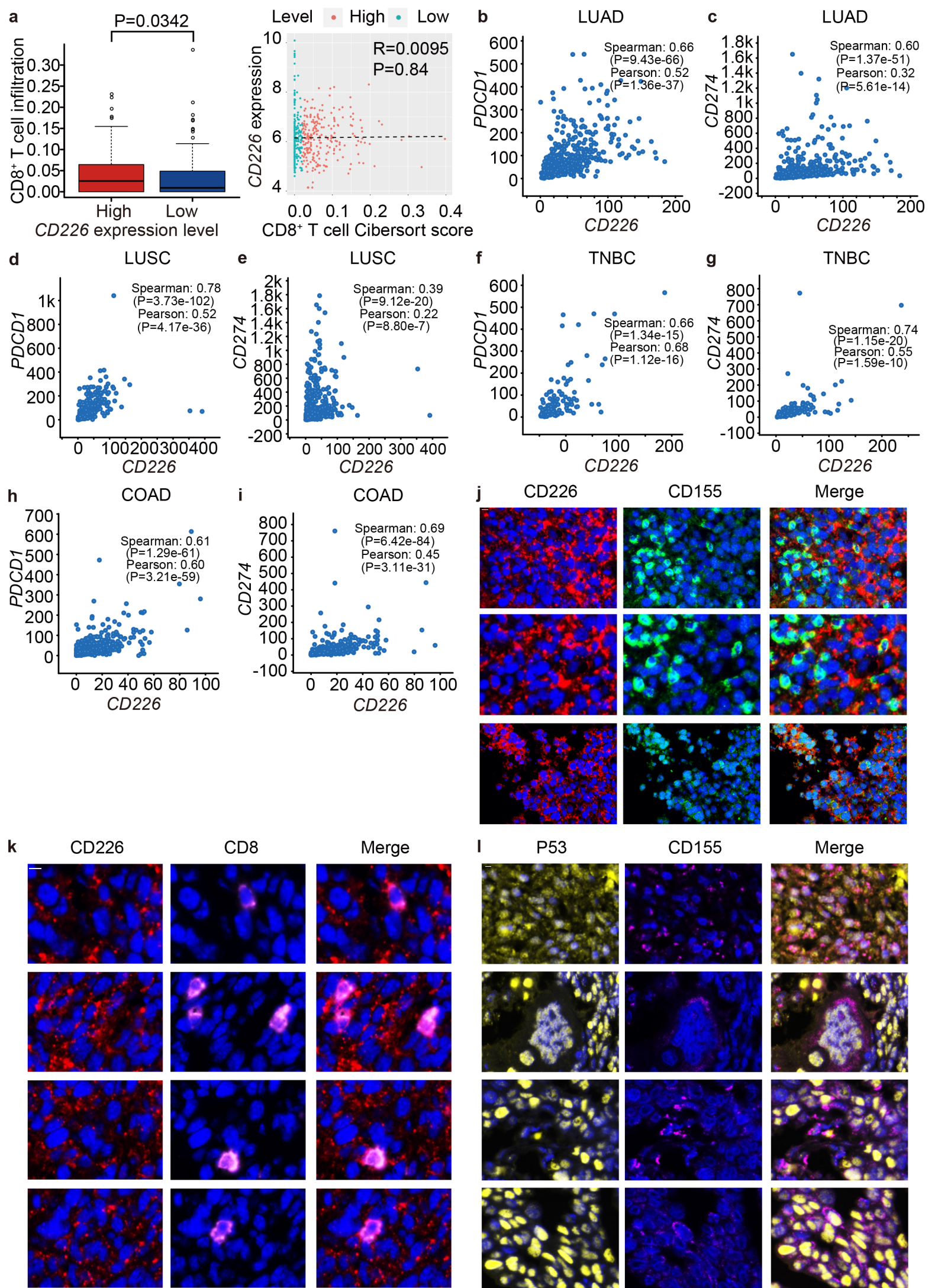

Supplementary Figure 5

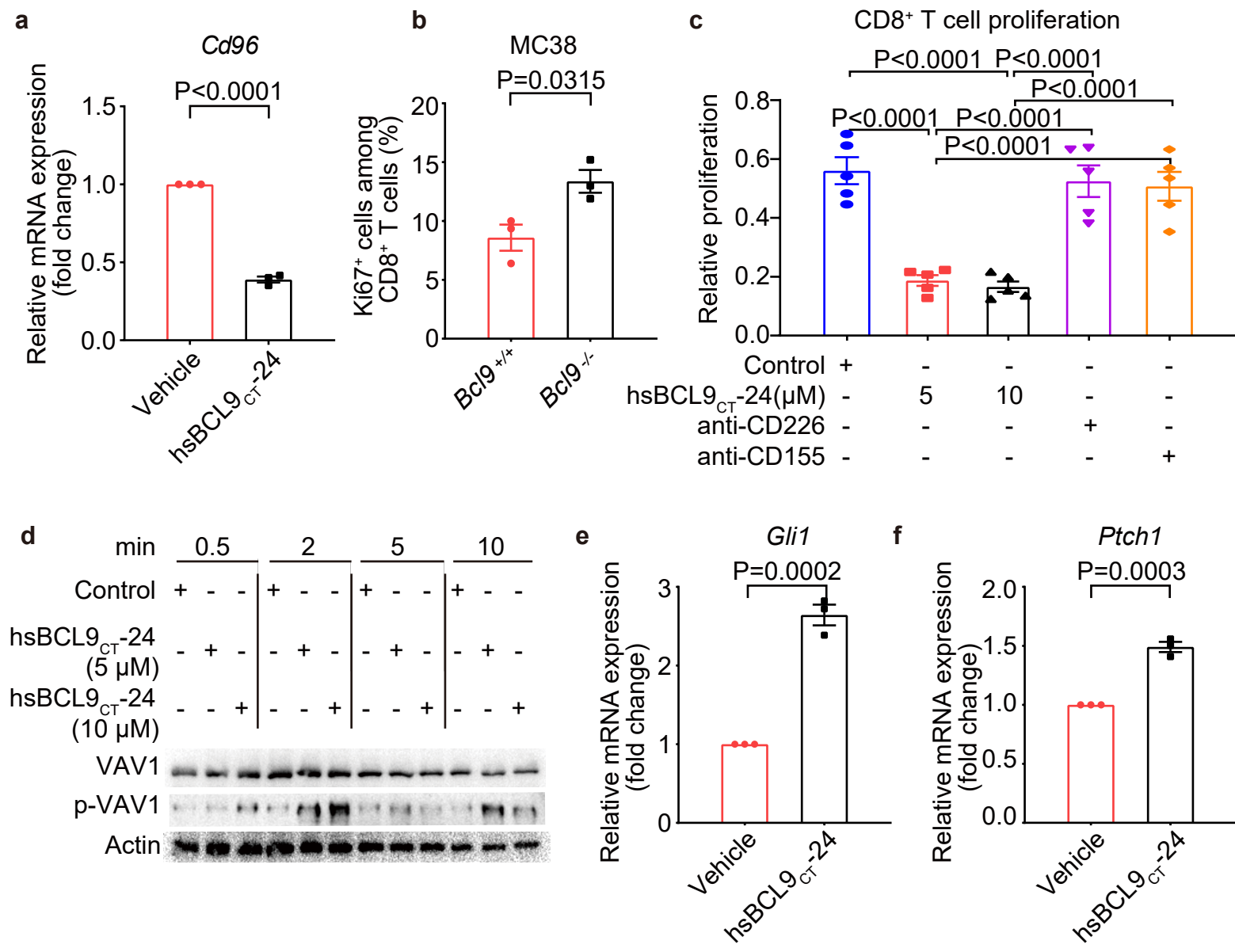

Supplementary Figure 6

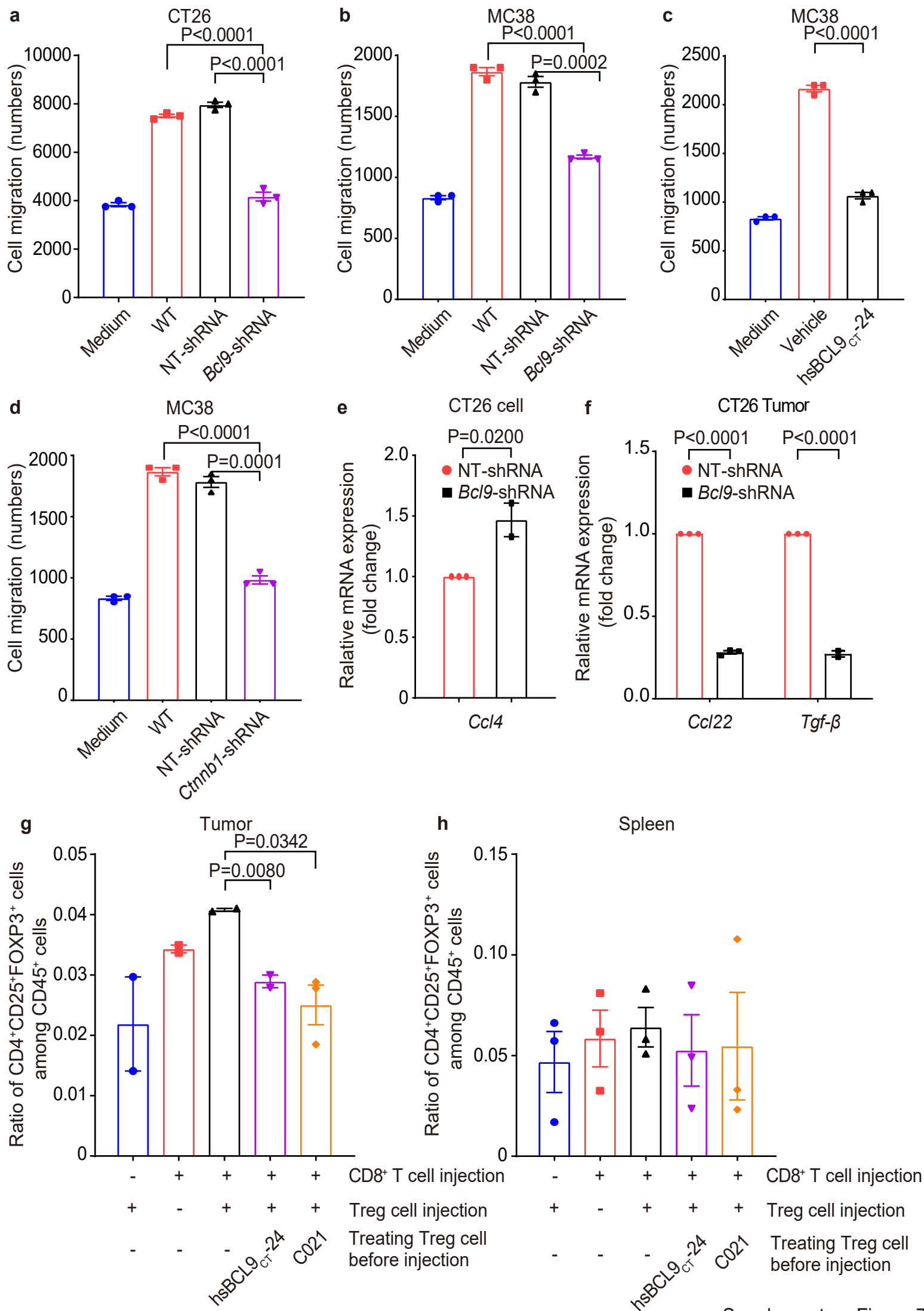

Supplementary Figure 7

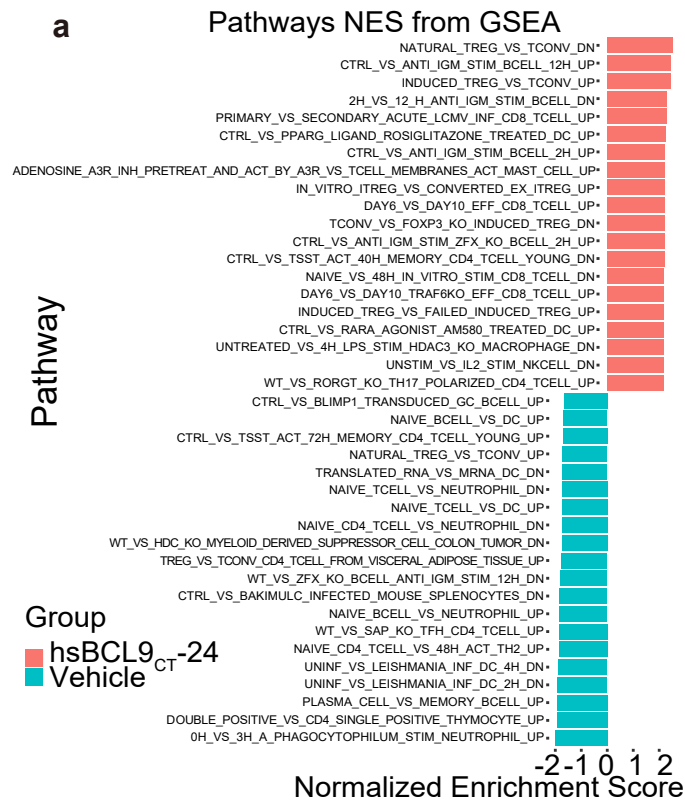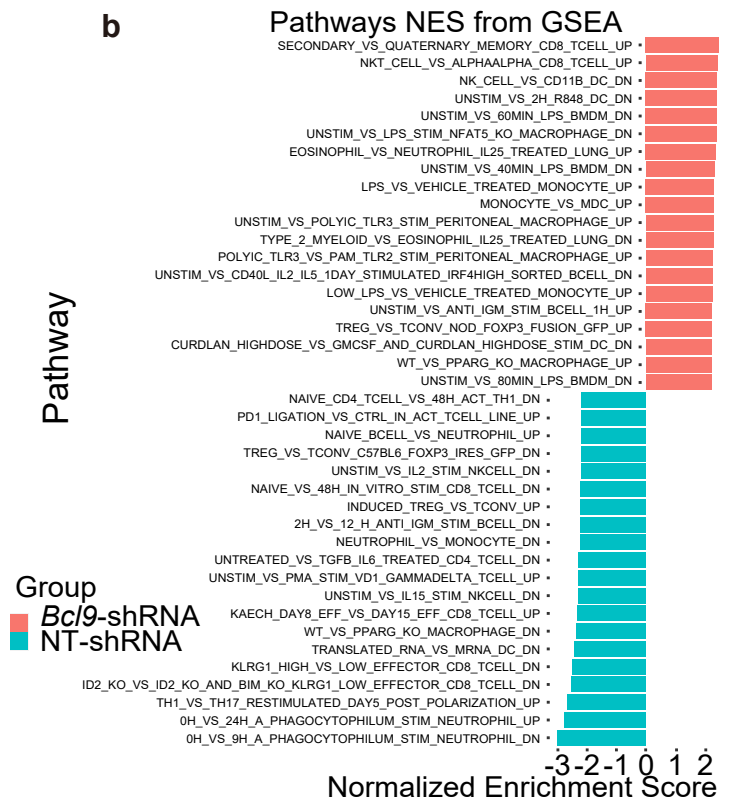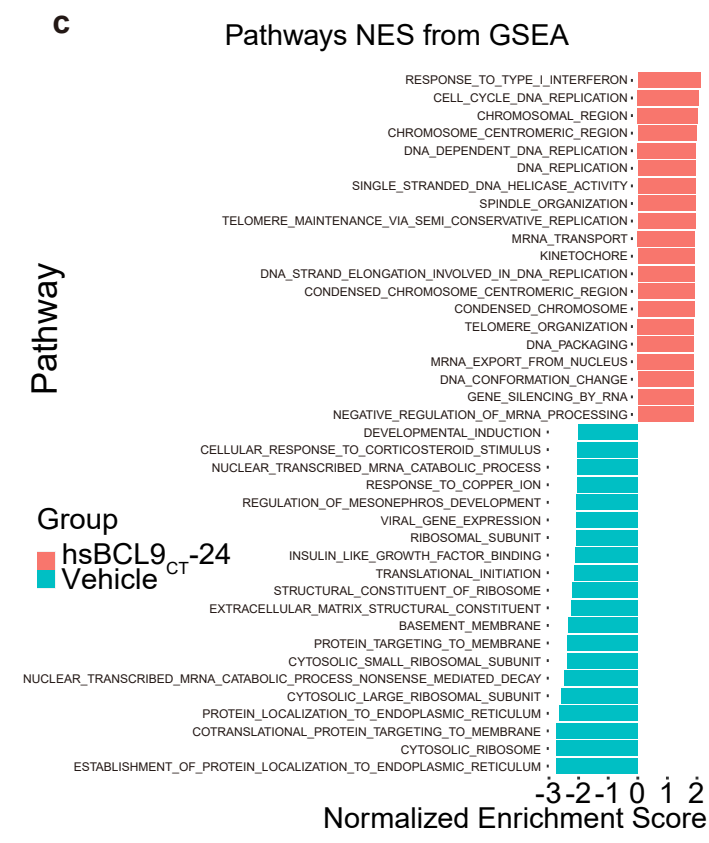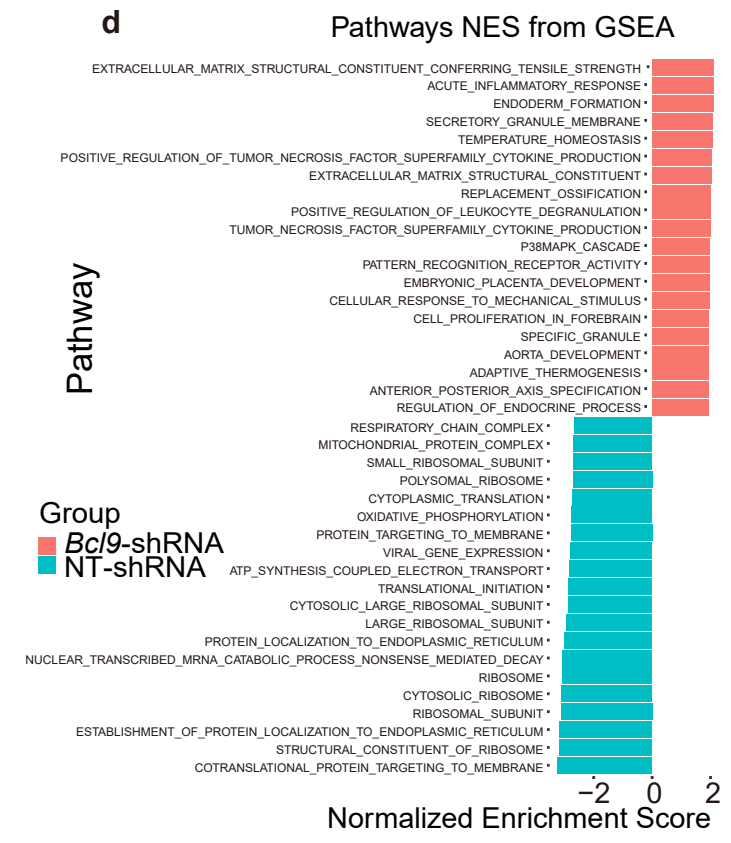

a

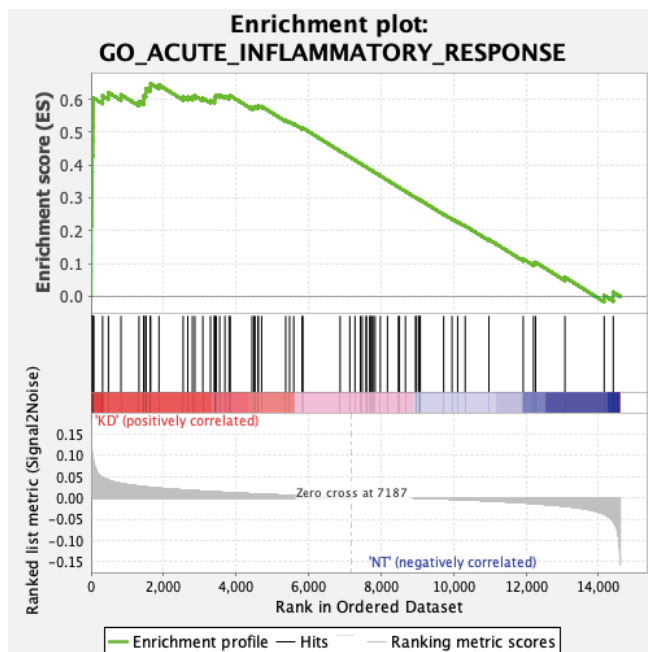

b

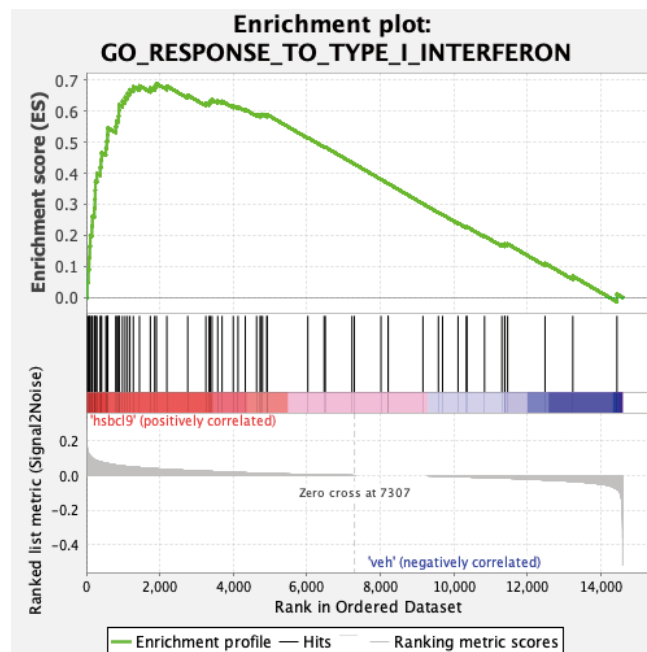

c

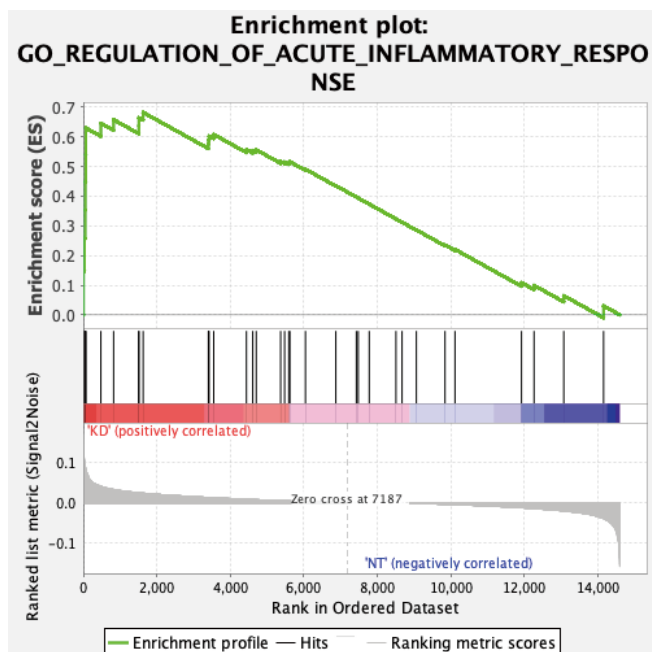

d

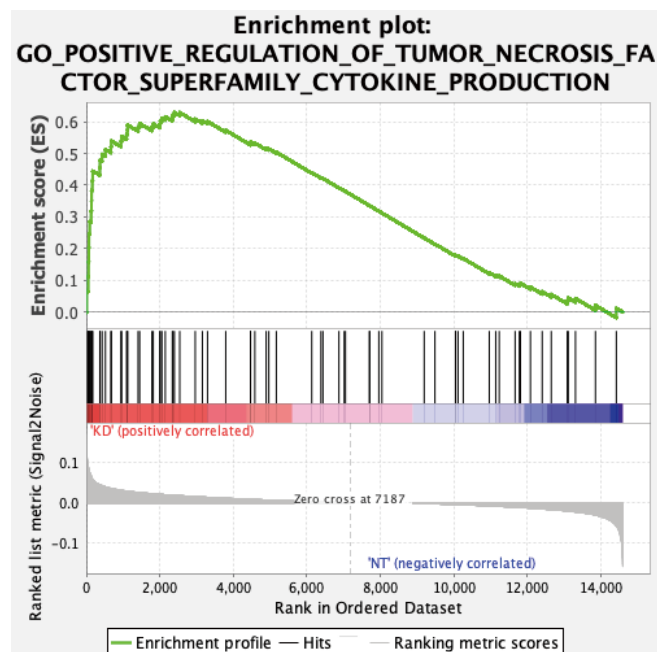

e

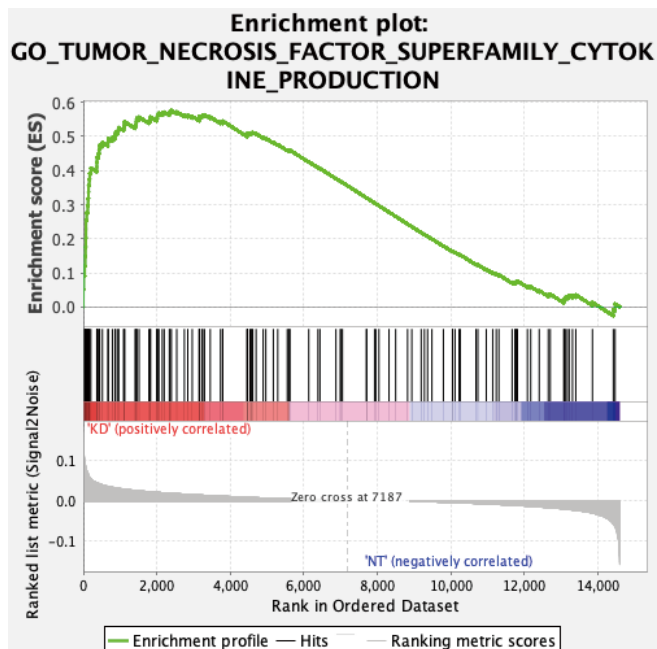

f

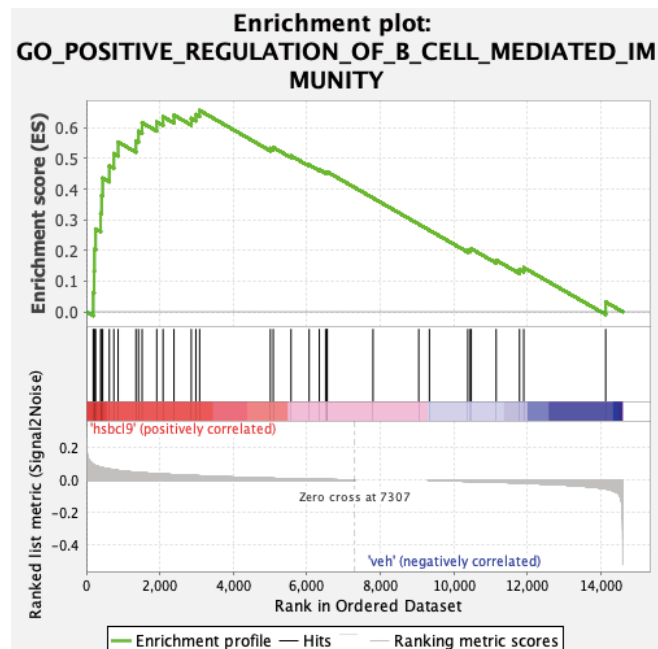

a

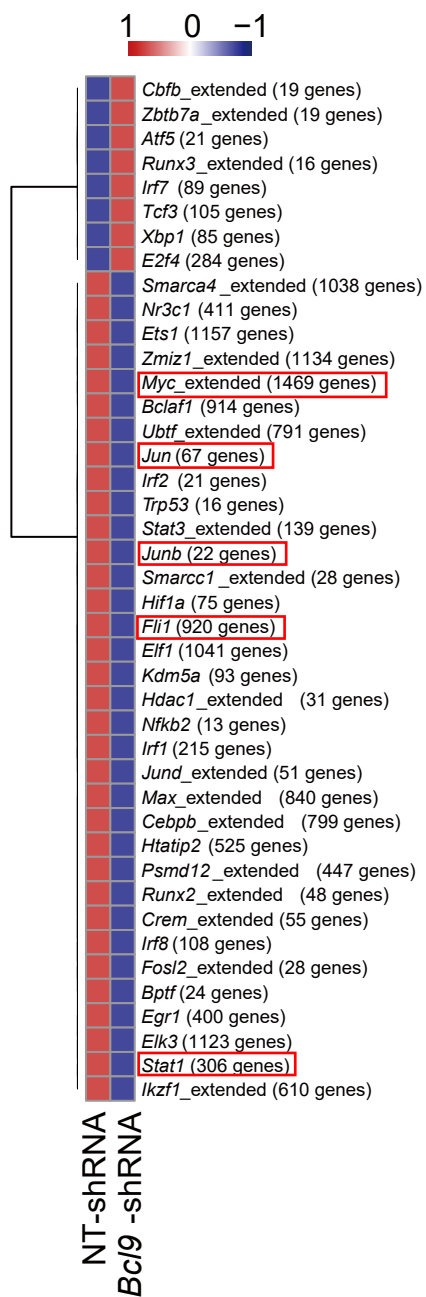

b

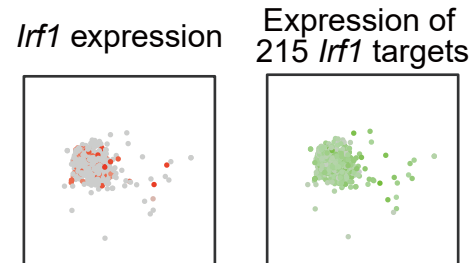

c

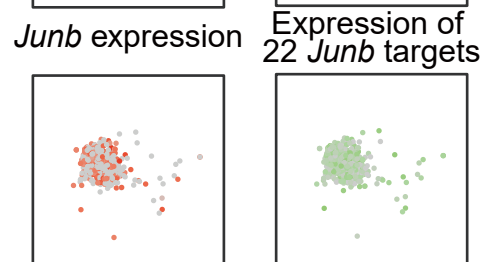

d

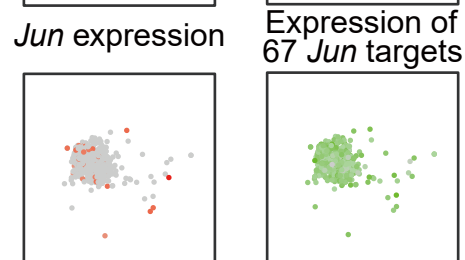

e

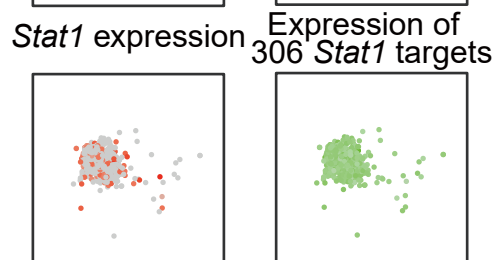

f

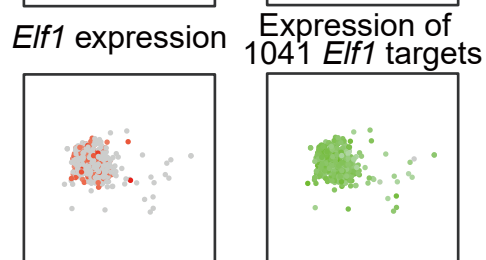

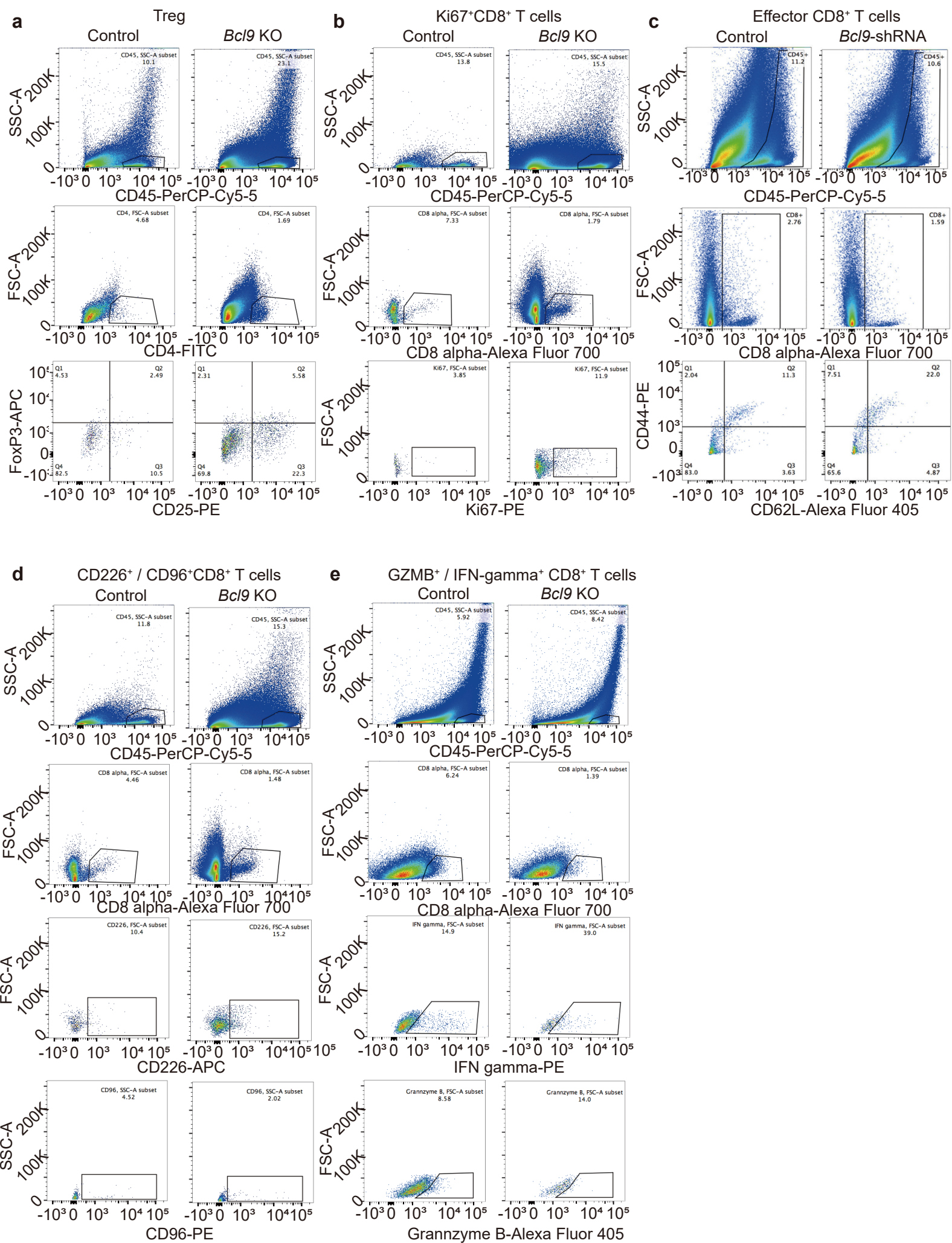

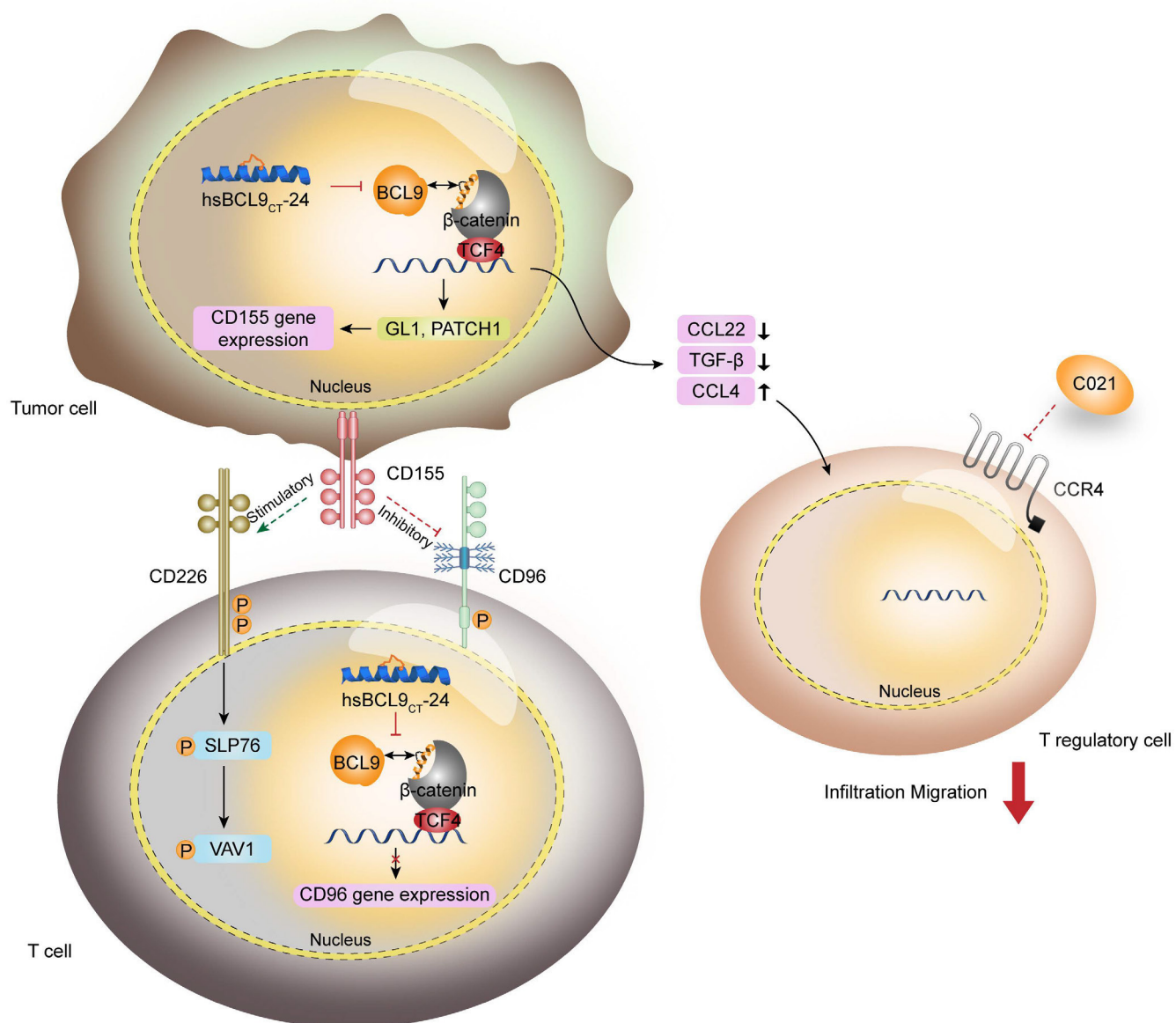

Supplement: Supplementary file 3 — Supplementary Figures [file 41392_2021_730_MOESM3_ESM.pdf]
